# (3+2)-Cycloadditions of Levoglucosenone (LGO) with Fluorinated Nitrile Imines Derived from Trifluoroacetonitrile: An Experimental and Computational Study †

**DOI:** 10.3390/molecules28217348

**Published:** 2023-10-30

**Authors:** Grzegorz Mlostoń, Katarzyna Urbaniak, Marcin Palusiak, Zbigniew J. Witczak, Ernst-Ulrich Würthwein

**Affiliations:** 1 Department of Organic & Applied Chemistry, Faculty of Chemistry, University of Lodz, Tamka 12, PL-91-403 Lodz, Poland; katarzyna.urbaniak@chemia.uni.lodz.pl; 2 Department of Physical Chemistry, Faculty of Chemistry, University of Lodz, Pomorska 163/165, PL-90-236 Lodz, Poland; marcin.palusiak@chemia.uni.lodz.pl; 3 Department of Pharmaceutical Sciences, Nesbitt School of Pharmacy, Wilkes University, 84 W. South Street, Wilkes-Barre, PA 18766, USA; zbigniew.witczak@wilkes.edu; 4 Organisch-Chemisches Institut and Center for Multiscale Theory and Computation (CMTC), Universität Münster, Corrensstrasse 40, D-48149 Münster, Germany

**Keywords:** levoglucosenone, nitrile imines, (3+2)-cycloadditions, fluoroalkylated pyrazoles, DFT calculations

## Abstract

The in situ-generated *N*-aryl nitrile imines derived from trifluoroacetonitrile smoothly undergo (3+2)-cycloadditions onto the enone fragment of the levoglucosenone molecule, yielding the corresponding, five-membered cycloadducts. In contrast to the ‘classic’ *C*(Ph),*N*(Ph) nitrile imine, reactions with fluorinated *C*(CF_3_),*N*(Ar) analogues lead to stable pyrazolines in a chemo- and stereoselective manner. Based on the result of X-ray single crystal diffraction analysis, their structures were established as *exo*-cycloadducts with the location of the *N*-Ar terminus of the 1,3-dipole at the α-position of the enone moiety. The DFT computation demonstrated that the observed reaction pathway results from the strong dominance of kinetic control over thermodynamic control.

## 1. Introduction

The (3+2)-cycloaddition reactions developed by R. Huisgen in the 1960s are recognized as one of the most general methods widely applied for the preparation of five-membered heterocyclic rings, which in many cases are of great importance for the manufacturing of drugs, agrochemicals, and a plethora of other practically useful organic materials [1,2,3,4]. In addition to the great practical importance of (3+2)-cycloaddition reactions, lively discussion on their mechanisms significantly contributed to the development of a general theory of the mechanisms of organic reactions in recent decades [2,3,4].

**Scheme 1:**
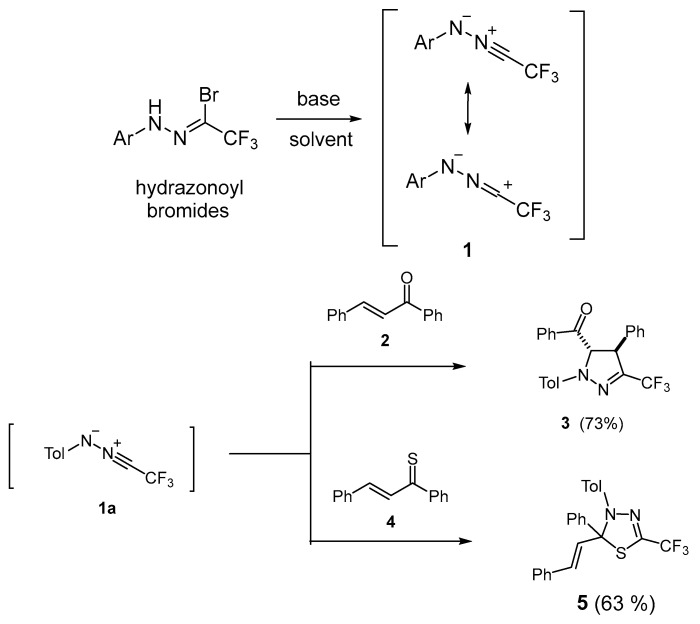
Generation of fluorinated nitrile imines **1** and two literature examples of their (3+2)-cycloadditions with chalcone **2** and thiochalcone **4**, leading to pyrazoline **3** and 1,3,4-thiadiazoline derivative **5**, respectively [7,14].

One of the most important and widely applied groups of so-called ‘*N*-centered 1,3-dipoles’ used in these reactions is nitrile imines, which are classified as ‘propargylic 1,3-dipoles’ [5,6,7,8]. In general, they cannot be isolated as stable compounds, but when generated in situ, they can be trapped with suitable dipolarophiles, yielding the corresponding (3+2)-cycloadducts. In recent years, fluorinated nitrile imines **1**, derived from trifluoroacetonitrile, have been readily available from hydrazonoyl halides by treatment with a base. They were widely useful tools for the preparation of bioactive, trifluoromethyl-substituted pyrazolines of type **3** by means of the (3+2)-cycloaddition with a suitable olefinic dipolarophile **2** (Scheme 1). Recently published results demonstrated that fluorinated nitrile imines **1** smoothly undergo (3+2)-cycloadditions not only with electron-rich but also with diverse electron-deficient olefinic dipolarophiles [9,10,11,12,13]. For example, nitrile imine **1a** reacts with chalcone **2**, yielding stereoselectively 3-trifluoromethyl-substituted pyrazoline **3** as an exclusive product in a good yield [12] (Scheme 1).

Moreover, some spirobispyrazolines were obtained through a double (3+2)-cycloaddition of nitrile imines **1** onto cumulated C=C bonds in alkoxyallenes [13].

Interestingly, the (3+2)-cycloaddition with sulfur analogues of chalcones, i.e., with thiochalcones of type **4**, occurred with inversed chemoselectivity along the C=S bond, and corresponding 1,3,4-thiadiazole derivatives of type **5** were obtained as exclusive products [14] (Scheme 1). In addition, diverse thioketones (aromatic, ferrocenyl, and cycloaliphatic) react as typical ‘superdipolarophiles’ [2] with nitryl imines **1**, yielding highly substituted analogues of heterocycles **5** in a regioselective manner [15,16] Electron deficient fluorinated thioamides and fluorinated nitroalkenes also react regioselectively, with **1** yielding highly fluorinated 2-trifluoromethyl-1,3-dihydro-1,3,4-thiadiazole and 3-trifluoromethyl-5-fluoropyrazole derivatives, respectively [17,18]. In a very recent publication, the synthesis of fused 3-trifluoromethyl-1,2,4-triazoles by means of a multi-step reaction of nitrile imines **1** with enolizable 2,3-dihydro-benzoimidazole-2-thiones was reported [19].

**Figure 1:**
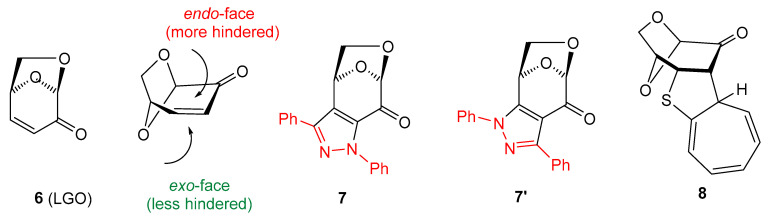
Levoglucosenone (**6**), its stereochemical structure, and selected products of cycloaddition reactions with a *C*(Ph),*N*(Ph) nitrile imine (**9**) and tropothione lead to polycylic, isomeric pyrazoles **7**/**7′** [30] and polycylic thiophene derivative **8** [39], respectively. In structures **7**/**7′**, the skeleton of *C*(Ph),*N*(PH) nitrile imine **9** is presented in red.

Levoglucosenone (LGO, **6**) (Figure 1), with an α,β-unsaturated ketone unit incorporated in its structure, is an exceptional carbohydrate derivative that offers unique opportunities for exploration in asymmetric synthesis as a chiral dipolarophile and/or dienophile. It was identified in 1970 as one of the products of acid-catalyzed cellulose pyrolysis [20]. Modified methods for manufacturing LGO, based either on low-temperature pyrolysis of microcrystalline cellulose or on fast pyrolysis of acid-impregnated cellulose, were described in recent publications [21,22]. The rapid development of LGO chemistry during the last five decades and its applications in medicinal and polymer chemistry are reported in review articles and original publications, which have regularly appeared in recent years [23,24,25,26,27,28,29]. Diverse transformations of LGO were achieved based, among others, on 1,3-dipolar cycloadditions [30,31,32], Diels-Alder reactions [33,34,35], and Michael additions [36,37,38]. For example, in a pioneering work, reactions of **6** with a ‘classic’, non-fluorinated benzonitrile imine **9** (*C*(Ph),*N*(Ph)) nitrile imine were reported to yield a mixture of two re-gioisomeric, fused pyrazoles **7** and **7′** (in a ca. 1:8 ratio), which were postulated to be formed as secondary products of a spontaneous oxidation of the initially formed (3+2)-cycloadducts, i.e., the corresponding pyrazolines; very likely, both compounds were formed as products of the preferred *exo* attack under the harsh reaction conditions (boiling toluene, 1.5 h heating) [30]. Moreover, in a very recent study, LGO **6** was shown to smoothly enter a ‘higher order (8+2)-cycloaddition’ with tropothione, yielding the hitherto unknown polycyclic thiophene derivative **8** [39].

Prompted by these results, we were motivated to check whether fluorinated nitrile imines **1** bearing differently substituted *N*-aryl moieties can undergo the anticipated (3+2)-cycloadditions onto the enone fragment of **6**, forming the pyrazoline ring attached to the levoglucosenone skeleton. From a mechanistic point of view, the regio- and diastereoselectivity of these cycloaddition reactions and the comparison with the results reported for nitrile imine **9** were also of interest.

## 2. Results and Discussion

### 2.1. Experimental Study

**Scheme 2:**
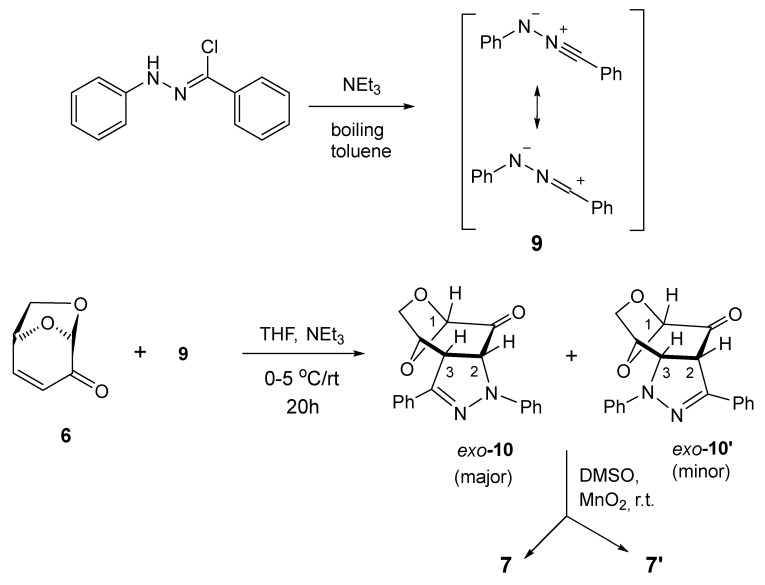
Selectivity was observed in the (3+2)-cycloaddition of *C*(Ph),*N*(Ph) nitrile imine (**9**) [7,30] with levoglucosenone (**6**) in THF solution at room temperature, providing pyrazolines *exo*-**10** and *exo*-**10′** as primary products, followed by oxidation to isolated pyrazole derivatives **7** and **7′**.

This study started with repetition of the already reported (3+2)-cycloaddition of **6** with benzonitrile *N*-phenylimine (*C*(Ph),*N*(Ph) nitrile imine) (**9**), but in contrast to the published protocol comprising the generation of the latter in boiling toluene [30], the reaction was performed in THF solution at room temperature using triethylamine as a base (Scheme 2).

**Figure 2:**
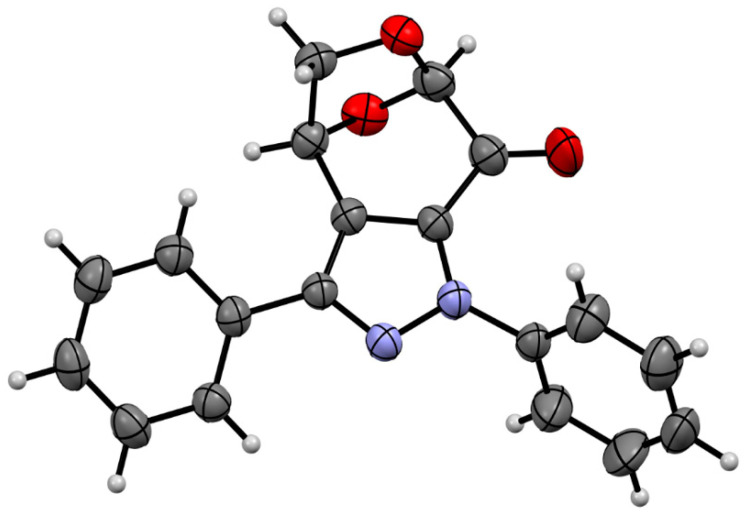
The molecular structure of polycylic pyrazole **7** was estimated by means of a single crystal X-ray experiment. Atoms are represented by thermal elipsoids (50%) for clarity.

After 24 h, the dipolarophile **6** was completely consumed, and the ^1^H NMR registered for the crude product revealed the presence of two regioisomeric (3+2)-cycloadducts, which showed characteristic signals assigned to the *H*C(3) and *H*C(2) atoms, located at the fused rings. They were found as doublets at 4.22/5.02 ppm (^3^*J*_H,H_ = 11.9 Hz) and 4.48/4.69 ppm (^3^*J*_H,H_ = 11.3 Hz), respectively, and comparison of the integration lines allowed to estimate the ratio of cycloadducts **10** and **10′** as ca. 75:25. All attempts to separate these isomeric compounds either by crystallization or column chromatography were unsuccessful. For this reason, they were oxidized by treatment with MnO_2_ in DMSO solution in an overnight experiment. After flash chromatography, a mixture of two products expected to be isomeric fused pyrazoles **7** and **7′** with characteristic absorptions of *H*C(1) atoms located at 5.57 ppm (s, major) and 5.59 ppm (s, minor), respectively, were found in the NMR spectrum. This time, the ratio of both isomers was calculated to be ca. 65:35. The obtained isomers were separated by preparative layer chromatography (PLC) on silica gel, and the major product was isolated as the less polar fraction. Crystallization from the petroleum ether/dichloromethane mixture gave single crystals suitable for the X-ray analysis, which unambiguously confirmed the structure of isomer **7** (Figure 2).

However, further attempts to grow suitable monocrystals of the purified minor isomer **7′** were unsuccessful. The described experiment allows us to conclude that the initial (3+2)-cycloaddition of **6** and **9** led to the formation of two regioisomeric cycloadducts **10** and **10′** with moderate regioselectivity, and this result confirms the previously reported observation [30]. However, our results unambiguously demonstrate that the structure of the major isomer **7** is the opposite of that suggested as the major isomer by the authors of the earlier study [30]. Based on the generally known tendency of LGO **6** to yield sterically favorable *exo*-cycloadducts, one can anticipate that this orientation of heterocyclic fragments has to be attributed to the initially formed fused pyrazolines *exo*-**10** (major) and *exo*-**10′** (minor). 

The test experiment with a representative of the fluorinated nitrile imines of type **1** was performed with the in situ-generated 1,3-dipole **1a**, starting with the corresponding hydrazonoyl bromide (Ar = 4-MeC_6_H_4_) and **6**. In this case, dichloromethane (and not THF or boiling toluene) was applied as a solvent at room temperature, and calcined K_2_CO_3_ served as a base. The progress of the reaction was monitored by TLC, and the experiment was stopped when no precursor **6** was detected in the reaction solution (in this case, after 18 h). After filtration of the inorganic salts and evaporation of the solvent, the crude mixture was examined by ^1^H NMR spectroscopy, which revealed the presence of only one cycloadduct with two characteristic signals of the levoglucosenone skeleton located at 5.28 (*H*(C-1), s) and 5.24 (*H*(C-2), d) ppm, and the singlet attributed to a methyl group at 2.34 ppm. The pure product was isolated by column chromatography in 54% yield as pale yellow crystals with m.p. Approximately 132–133 °C and tentatively identified as the expected (3+2)-cycloadduct *exo*-**11a** with the three singlet signals registered along with multiplets attributed to the levoglucosenone skeleton and an *AB*-system of the p-tolyl ring located at 7.09 and 7.13 ppm (^3^*J*_H,H_ = 8.5 Hz). In the ^13^C NMR spectrum, the characteristic absorption of the CF_3_ group was found as a quartet (^1^*J*_C,F_ = 278 Hz) at 120.9 ppm. The anticipated molecular formula, C_15_H_13_F_3_N_2_O_3_, was confirmed by the correct elemental analysis.

**Figure 3:**
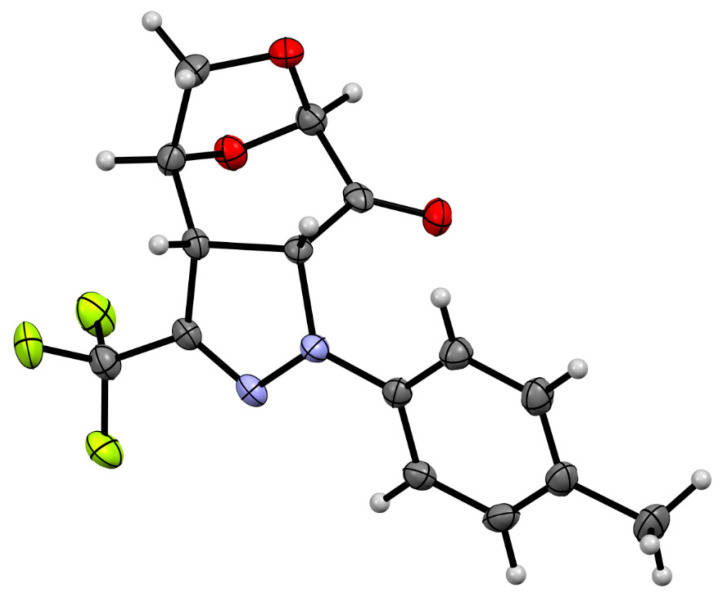
Molecular structure of polycylic pyrazoline *exo*-**11a**, estimated by means of a single crystal X-ray experiment. Atoms are represented by thermal elipsoids (50%) for clarity.

In order to determine the constitution and configuration of this product, single crystals were grown from MeOH, and the X-ray analysis revealed the structure presented in Figure 3.

**Scheme 3:**
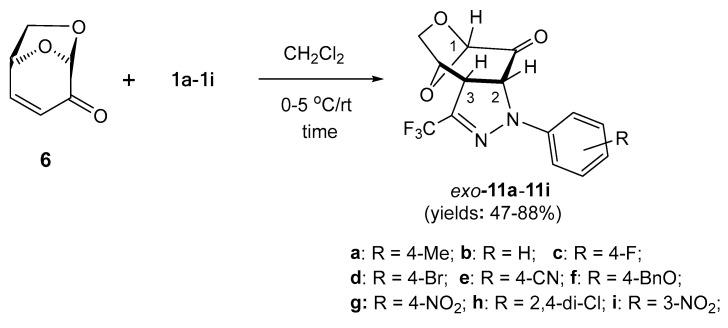
Highly selective (3+2)-cycloadditions of fluorinated nitrile imines **1a**–**1i**, derived from trifluoroacetonitrile, with levoglucosenone (**6**) leading to trifluoromethyl substituted, fused pyrazolines **11a**–**11i**.

It confirmed the formation of cycloadduct *exo*-**11a** in a regioselective manner with the new C–N bond connecting the α-carbon atom of the enone-part of **6** with the terminal *N*-atom of the 1,3-dipole framework. Thus, the regioselectivity of the (3+2)-cycloaddition corresponds to those observed in the reaction of **6** with *C*(Ph),*N*(Ph) nitrile imine **9**, leading to the major isomer *exo*-**10**. However, whereas **9** and **6** provide a mixture of regioisomeric cycloadducts, the 1,3-dipole **1a** undergoes the (3+2)-cycloaddition with complete regioselectivity. In addition, the X-ray analysis unambiguously indicates that the favored (as anticipated) *exo*-isomer **11a** was the only cycloadduct formed in the course of the (3+2)-cycloaddition of **6** with fluorinated nitrile imine **1a** (Scheme 3). This stereochemical aspect observed in the (3+2)-cycloadditions of **6** with nitrile imines **1** will be discussed in the part ‘Computational study′, which is based on the results obtained by using DFT calculations.

**Scheme 4:**
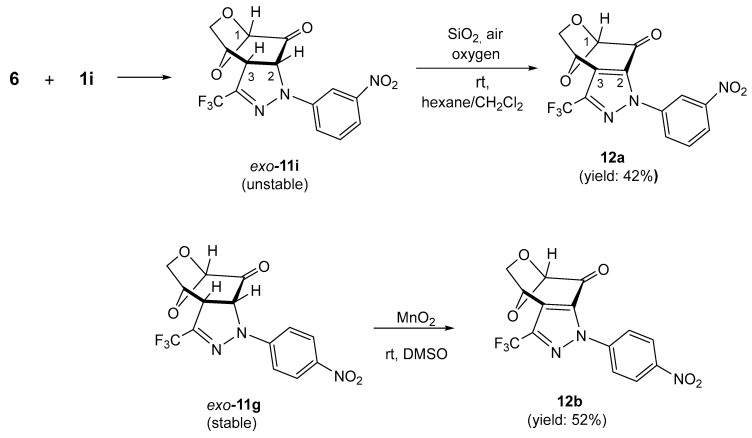
Equation above: spontaneous oxidation of pyrazoline *exo*-**11i** with air oxygen during chromatographic purification, leading to the fused pyrazole **12a**. Equation below: dehydrogenation of pyrazoline *exo*-**11g** using MnO_2_ as an oxidizing reagent, leading to the fused pyrazole **12b**.

Prompted by the high selectivity observed in the experiment with 1,3-dipole **1a**, we decided to test a series of similar (3+2)-cycloadditions starting with **6** as a reactive dipolarophile and differently substituted nitrile imines **1b**–**1i**. Under analogous reaction conditions, in all reactions, the expected tricyclic pyrazolines *exo*-**11b**–**11i** were obtained as sole products and subsequently isolated chromatographically in fair to good yields (47–88%) as stable, solid materials with the tendency to crystallize from the corresponding organic solvents (Scheme 3). In analogy to *exo*-**11a**, for all obtained products **11b**–**11i**, the structure of the anticipated *exo*-cycloadducts was also attributed based on the similarity of their spectroscopic properties (^1^H NMR and ^13^C NMR). Notably, the spontaneous oxidation of pyrazolines such as **10**/**10′** to the corresponding pyrazoles was not observed in most cases of trifluoromethyl-substituted cycloadducts **11**, and an exceptional behavior was observed only in the case of pyrazoline **11i**, when nitrile imine **1i** bearing Ar = *m*-NO_2_C_6_H_4_ was employed as the reactive 1,3-dipole. In the ^1^H NMR spectrum of the crude mixture, a characteristic doublet attributed to *H*C(2) as well as a singlet signal characteristic for *H*C(1) in the anticipated cycloadduct *exo*-**11i** were registered at 5.28 (^3^*J*_H,H_ = 11.7 Hz) and 5.21 ppm, respectively. In addition, a tiny singlet was also observed at 5.61 ppm, indicating the presence of an unknown, minor product. To our surprise, after column chromatography, the initially observed doublet disappeared and the later singlet with integration corresponding to one H-atom in the structure of the oxidized pyrazole **12a** at 5.61 ppm was present. Finally, this component was isolated by column chromatography as the major product in 42% yield (Scheme 4, above).

The collected spectroscopic data allowed us to determine the expected structure **12a**. For example, in the ^13^C NMR, weak absorptions of *C*(2) and *C*(3) were localized at 138.7 and 129.4 ppm, respectively. The signal of C=O was found at 178.8 ppm, and the characteristic quartet of CF_3_ with ^1^*J*_C,F_ = 260.7 Hz was found at 120.5 ppm.

In extension of this study, a sample of the stable cycloadduct *exo*-**11g**, bearing an Ar = 4-NO_2_C_6_H_4_ substituent, was oxidized by treatment with MnO_2_ in DMSO solution based on the procedure applied for the mixture of isomeric pyrazolines **10**/**10′**. After 24 h, the expected pyrazole **12b** was isolated as a pure material with a 52% yield (Scheme 4, equation below). The reason for the particular sensitivity of pyrazoline *exo*-**11i** bearing the 3-NO_2_C_6_H_4_ substituent at the terminal N-atom of the 1,3-dipole to air oxygen is not known at the moment. 

**Scheme 5:**
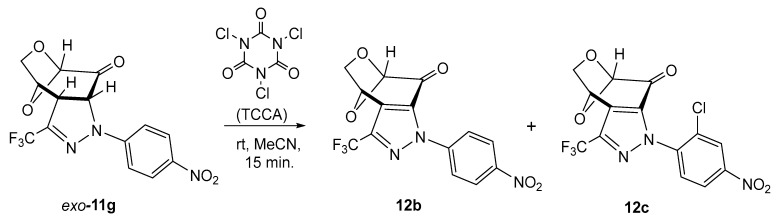
Attempted oxidation of pyrazoline *exo*-**11g** with TCCA, leading to a mixture of fused pyrazole **12b** and its chlorinated derivative **12c**.

Prompted by the results described in a recent study [40], we decided to check the oxidation of the stable pyrazoline *exo*-**11g** with trichloroisocyanuric acid (TCCA) (1.5 mol-equiv.) in acetonitrile solution. A series of experiments performed with different ratios of both substrates demonstrated that, irrespective of the reaction conditions, in all cases, mixtures of the above-described fused pyrazole **12b** and its chlorinated derivative **12c** were obtained in a ratio of ca. 85:15. The attempted separation of the mixture, either by crystallization or by standard column chromatography, was unsuccessful, and for this reason, both products, i.e., **12b** and **12c**, were identified spectroscopically in the mixture. This result allowed us to conclude that in the case of fused pyrazolines **11**, TCCA acts not only as an oxidant but also as a powerful chlorinating reagent, and the reaction leads to an undesired side product (Scheme 5). This observation fits well with the results described in the above-cited publication [40].

The structure of the chlorinated derivative can tentatively be postulated as **12c**, which is suggested by all the analyzed signals found in the ^1^H NMR spectrum. Thus, three signals attributed to protons of the aromatic ring were found at 5.80 (dd), 7.97 (dd), and 8.41 (d) ppm, respectively. 

In a supplementary experiment aimed at comparing the ability of pyrazolines *exo*-**10**/*exo*-**10′** and *exo*-**11g** to undergo air oxidation, a sample of the latter compound was heated in boiling toluene under reflux for 1.5h. After this time, the obtained material was analyzed by running the ^1^H NMR spectrum, which demonstrated the presence of both compounds, starting with pyrazoline *exo*-**11g** and target pyrazole **12b** in a ratio of ca. 80:20. The oxidation of trifluoromethyl-substituted pyrazolines of type **11** occurs definitively slower than in the case of the known Ph-substituted analogues **10** [30].

**Figure 4:**
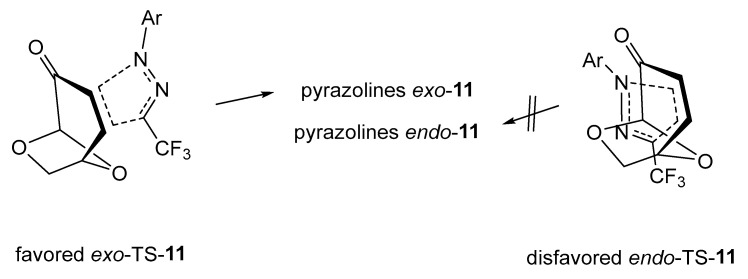
Competitive *exo*- and *endo*-approaches of nitrile imines **1** onto the enone fragment of levoglucosenone **6** in the transition state of the (3+2)-cycloaddition reaction.

A general mechanistic presentation of the studied (3+2)-cycloadditions of trifluoromethyl-substituted 1,3-dipoles **1** with levoglucosenone **6** is outlined in Figure 4. The sterically favored *exo-*transition state leads to the formation of tricyclic pyrazolines *exo*-**11** as exclusive products of these reactions. The approach of the 1,3-dipole from the *endo*-face of **6** is hindered due to the sterically more demanding -O-CH_2_- moiety compared to the -O- bridge. (See the computational part below).

The experiments presented above demonstrate that the (3+2)-cycloadditions of fluorinated nitrile imines **1** derived from trifluoroacetonitrile and the non-fluorinated *C*(Ph),*N*(Ph) nitrile imine **9** lead to pyrazolines *exo*-**11** and *exo*-**10/10′**, respectively, with high diastereoselectivity but with different regioselectivity. These observations prompted us to shed more light on the pathways of these concerted processes depicted in Figure 4. A detailed DFT calculation related to these problems will be discussed in the following part. 

### 2.2. Computational Study

#### Mechanistic Investigations by DFT Calculations

To understand the observed stereochemistry of the cycloaddition reactions, the experimental results will be discussed on the basis of the calculated Gibbs free energy surface (ΔG_298_ [kcal/mol]) as investigated by a comprehensive quantum chemical DFT study. The determination of kinetic and thermodynamic properties will be the main focus of this study.

Starting with B3LYP/6-31G(d) [41,42] + GD3BJ [43,44], geometry optimizations for the gas phase and PBE1PBE/def2tzvp [45,46,47,48] + GD3BJ optimizations for the PCM solvent sphere of dichloromethane [49] were performed. For all calculations, the GAUSSIAN 16 package of programs was used (see in Supplementary Materials) [50]. In accordance with the reaction conditions (room temperature, no irradiation), only closed shell calculations were performed, although diradical intermediates principally cannot be excluded (see Haberhauer, Gleiter et al. [51], Firestone [52], and also the comments given in ref. [2,3]). Haberhauer, Gleiter et al. [51] have convincingly shown that 1,3-dipolar cycloadditions of the nitrile oxides, which are quite similar to nitrile imines, involve alkenes in preferentially closed shell transition states, whereas alkynes may also involve diradical intermediates. In the following, we discuss differences in Gibbs free energies (ΔG_298_) [kcal/mol] (see also Supplementary Materials for details); all relative energies refer to the sum of the starting materials.

**Figure 5:**
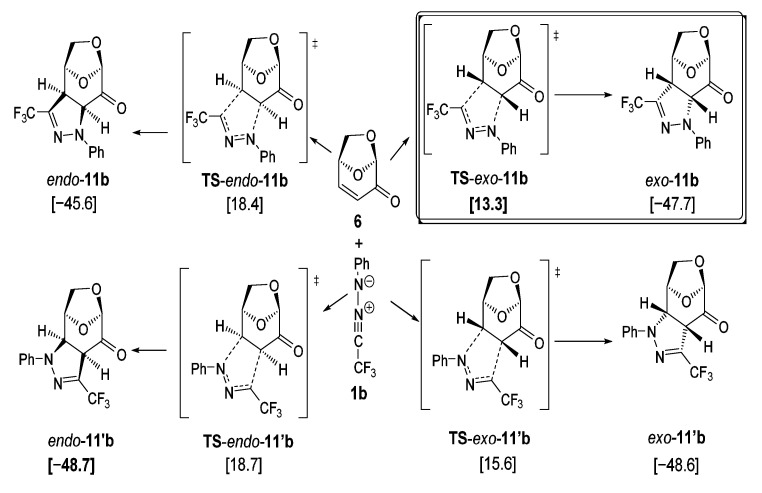
DFT calculations of the (3+2)-cycloadditions of trifluoromethyl-substituted nitrile imine **1b** with levoglucosenone **6** (PBE1PBE/def2tzvp + GD3BJ +PCM-dichloromethane) [kcal/mol] led to the regioisomeric pyrazolines *exo*-**11b**/*endo*-**11b** and *exo*-**11′b**/*endo*-**11′b**. Symbol${\;}^{‡}$: transition state.

For the calculations of the reaction leading to pyrazoline *exo***-11b** and its hypothetical isomers (Figure 5), the configurations and conformations were derived from the X-ray structure of *exo***-11a** (Figure 3). The transition states were localized by reaction path calculations elongating the respective C–C and C–N bonds stepwise, followed by complete geometry optimization. 

**Figure 6:**
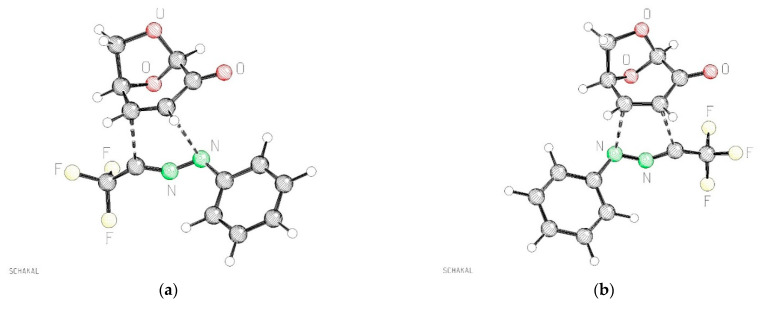
Calculated atomic distances of the forming bonds of the transition states **TS-***exo***-11b** and **TS-***exo***-11′b** (PBE1PBE/def2tzvp + GD3BJ +PCM-dichloromethane). (**a**): **TS-***exo***-11b** atomic distances C–N 2.569 Å, C–C 2.253 Å; (**b**): **TS-***exo***-11′b** atomic distances C–N 2.323 Å C–C 2.353 Å.

As shown in Figure 5, the trifluoromethyl-phenyl substituted nitrile imine **1b** was employed as the standard model for the 1,3-dipolar cycloadditions with LGO **6** used in the experiments. As the calculations show, all four cycloadditions proceed very exothermically (−45.6 to −48.7 kcal/mol). However, the calculated kinetic barriers (Gibbs free activation energies) are quite different, with **TS-***exo-***11b** [13.3 kcal/mol] being the significantly lowest transition state calculated. This transition state leads to the experimentally observed product *exo-***11b**, which is not the thermodynamically best isomer. Thus, this reaction is a good example of a kinetically controlled process leading exclusively to the experimentally observed stereoisomer *exo-***11b**. As shown in Figure 4, the 1,3-dipole approaches **6** from the sterically less hindered face (lower side, according to the drawings presented in Figure 5 and Figure 6), leading to the *exo*-product. The alternative face of **6** is sterically more hindered due to the presence of the bulkier CH_2_O- moiety. The *exo*-transition states are found to be lower by ~5 kcal/mol (Figure 5 and Figure 6) compared to the *endo*-transition states. Significantly shorter distances between the O-CH_2_-group and the 1,3-dipole lead to much higher repulsion for the *endo*- compared to the *exo*-transition states. Thus, the expected diastereoselectivity is nicely reflected by the DFT calculations.

Concerning the structural properties of the two lowest transition states, **TS-***exo-***11b** and **TS-***exo-***11′b**, significant geometrical differences were found, as illustrated in Figure 6. While for the lower transition state **TS-***exo-***11b** quite different atomic distances between the reacting carbon and nitrogen atoms were observed (C–N 2.569 Å, C–C 2.253 Å), illustrating the fairly unsymmetrical formation of the new bonds, the second-best transition state **TS-** *exo-***11′b** shows quite similar distances between the reacting atoms: C–N 2.323 Å C–C 2.353 Å. The two other possible approaches of the nitrile imine **1b** to the alternative face of **6** show significantly higher barriers (18.4 to 18.7 kcal/mol). The hypothetical product *endo-***11′b**, resulting from the transition state **TS-***endo-***11′b**, would be one of the thermodynamically best isomers of all possible products, but it seems to be kinetically not easily accessible.

**Figure 7:**
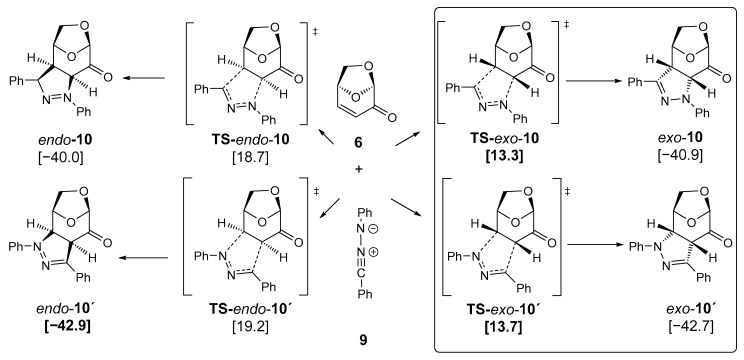
DFT calculations of the cycloadditions of diphenyl-substituted nitrile imine **9** with levoglucosenone **6** (PBE1PBE/def2tzvp + GD3BJ +PCM-dichloromethane, room temperature) [kcal/mol] led to diastereomeric and regioisomeric pyrazolines *exo*/*endo*-**10** and *exo*/*endo*-**10′**. Symbol${\;}^{‡}$: transition state.

For comparison, quantum chemical calculations were also performed for the corresponding 1,3-dipolar cycloaddition reaction of diphenylnitrilimine **9** [53,54,55] with LGO **6**. As shown in Figure 7, the results are mostly similar to the data of the CF_3_-substituted nitrilimine **1b**, with a remarkable difference between the transition state energies of **TS-***exo-***10** and **TS-***exo-***10’**. Both activation energies are low but quite similar, thus explaining the experimental observation of both diastereomeric products, *exo-***10** and *exo-***10’** (Scheme 2). Again, the *endo-*isomers are not accessible due to higher barriers. Kinetic control determines the constitution of the products observed, whereas thermodynamics is less important for the reaction products.

In summary, the computational investigations presented here interpret the experimental findings as the result of the strong dominance of kinetic control over thermodynamic control.

## 3. Materials and Methods

CCDC Deposition: see Table S1 (see in Supplementary Materials).

**General information:** Chemicals, hexane, and petroleum ether were purchased and used as received without further purification. Other solvents (CH_2_Cl_2_, THF) were purified by distillation prior to their usage. Crude products (reaction mixtures) were purified by standard preparative layer chromatography (PLC) on plates coated with silica gel (60 Å medium pore diameter, PF_254_, Merck) or alternatively by standard column chromatography (silica gel, 40 mesh, J. T. Baker)). Unless stated otherwise, yields refer to amounts of isolated products.

Melting points were determined in capillaries with a Stuart SMP30 apparatus with automatic temperature monitoring and are uncorrected. The NMR spectra were recorded with a Bruker Avance III 600 MHz instrument (^1^H NMR: 600 MHz; ^13^C NMR: 151 MHz). Chemical shifts are reported relative to solvent residual peaks (^1^H NMR: δ = 7.26 ppm [CHCl_3_]; ^13^C NMR: δ = 77.0 ppm [CDCl_3_]). IR spectra were run on the Agilent Cary 630 FTIR spectrometer. ESI-MS measurements were performed with a Varian 500-MS LC Ion Trap instrument. High-resolution mass spectrometry (HRMS) measurements were performed using a Synapt G2-Si mass spectrometer (*Waters*) equipped with an ESI source and quadrupole-time-of-flight mass analyzer. Optical rotations were determined with an Anton Paar MCP 500 polarimeter at the temperatures indicated. Elemental analyses were obtained with a Vario EL III (*ElementarAnalysensysteme GmbH*) instrument. 

**Starting materials:** Levoglucosenone (**6**) was prepared from cellulose by sulfuric acid-assisted pyrolysis, following a known procedure [56]. Hydrazonoyl bromides used as precursors of the in situ-generated nitrile imines **1** were synthesized by bromination of corresponding trifluoroacetaldehyde arylhydrazones, prepared in the presence of molecular sieves, according to a known procedure [57], using NBS as a brominating reagent [14]. 

**Reactions of nitrile imines 1a**–**1b with levoglucosenone** (**6**). *General procedure*: Approximately 1 mmol of **6** and 1.1 mmol of the corresponding hydrazonoyl bromide were dissolved in 2–3 mL of dry dichloromethane, and an excess of freshly dried K_2_CO_3_ was added. The mixture was magnetically stirred until **6** was completely consumed (24–72 h). Upon completion of the reaction, potassium carbonate was filtered off, and, after evaporation of the solvent in a vacuum evaporator, the crude mixtures were purified by column chromatography using petroleum ether with increasing amounts of methylene chloride as an eluent. Analytically pure samples were obtained after crystallization of the product in petroleum ether with a small amount of dichloromethane.

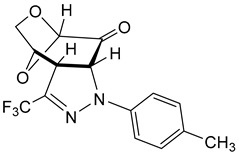

*5-(p-Tolyl)-3-(trifluoromethyl)-9,11-dioxa-4,5-diazatricyclo[6.2.1.02,6]undec-3-en-7-one* (*exo*-**11a**). Yield: 175 mg (54%), pale-yellow crystals, mp = 132–133 °C (petroleum ether/CH_2_Cl_2_). [α]_D_^20^ = –629 (*c* 0.3, CHCl_3_).
^1^H NMR (CDCl_3_): δ 2.34 (*s*, 3H, CH_3_); 4.01 (*d*, *J*_H,H_ = 12.2 Hz, 1H); 4.07–4.09 (*m*, 1H); 4.12–4.14 (*m*, 1H); 5.15 (*d*, *J*_H,H_ = 4.7 Hz, 1H); 5.24 (*d*, *J*_H,H_ = 12.1 Hz, 1H); 5.28 (*s*, 1H); 7.09, 7.13 (*AB*-system, *J*_H,H_ = 8.5 Hz, 4CH_arom_);
^13^C NMR (CDCl_3_): δ 21.1, 51.8, 63.6, 70.9, 100.2, 68.6, 114.5, 129.9, 120.9 (*q*, *^1^J*_C,F_ = 277.7 Hz, C*F*_3_); 131.6, 140.7, 134.0 (*q*, ^2^*J*_C,F_ = 36.9 Hz, *C*–CF_3_), 194.5 (C=O);
^19^F NMR: δ –63.9;
IR: ν 1703*s* (C=O), 1510*s*, 1378*m*, 1299*m*, 1258*s*, 1193*m*, 1123*vs*, 1083*s*, 943*m*, 801*s*, cm^−1^;
EA for C_15_H_13_F_3_N_2_O_3_ (326.28): calcd. C 55.22, H 4.02, N 8.59; found C 55.03, H 4.04, N 8.85.

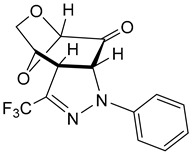

*5-Phenyl-3-(trifluoromethyl)-9,11-dioxa-4,5-diazatricyclo[6.2.1.02,6]undec-3-en-7-one* (*exo*-**11b**). Yield: 190 mg (61%), pale yellow crystals, mp = 108–109 °C (petroleum ether/CH_2_Cl_2_). [α]_D_^20^ = –515 (*c* 0.3, CHCl_3_).
^1^H NMR (CDCl_3_): δ 3.96 (*dd*, *J*_H,H_ = 12.0 Hz, *J*_H,H_ = 1.0 Hz, 1H); 4.04–4.07 (*m*, 1H); 4.09–4.10 (*m*, 1H); 5.13 (*d*, *J*_H,H_ = 2.3 Hz, 1H); 5.24 (*d*, *J*_H,H_ = 11.9 Hz, 1H); 5.26 (*s*, 1H); 7.00–7.03 (*m*, 1CH_arom_); 7.17–7.20 (*m*, 2CH_arom_); 7.30–7.34 (*m*, 2CH_arom_);
^13^C NMR: δ 51.6, 63.2, 71.1, 100.4, 68.5, 114.4, 121.9, 129.2, 120.8 (*q*, *^1^J*_C,F_ = 268.1 Hz, C*F*_3_); 134.8 (*q*, *^2^J*_C,F_ = 37.3 Hz, *C*–CF_3_); 142.9, 194.6 (C=O).
^19^F NMR (CDCl_3_): δ –64.0;
IR: ν 1744*s* (C=O), 1600*s*, 1494*s*, 1312*s*, 1297*m*, 1259*s*, 1185*s*, 1092*vs*, 976*s*, 913*s*, 799*s*, 752*s*, cm^−1^;
EA for C_14_H_11_F_3_N_2_O_3_ (326.28): calcd. C 53.85, H 3.55, N 8.97; found C 53.78, H 3.54, N 9.22.

## 4. Conclusions

In recent years, fluorinated and fluoroalkylated pyrazole derivatives have attracted great attention as biologically active compounds, and some representatives have already found practical applications as bioactive components of some drugs and agrochemicals offered on the market, e.g., Celecoxib (Pfizer), Penflufen (Bayer), Isoflucypram (Bayer), etc. [58,59,60,61,62]. On the other hand, levoglucosenone (**6**) is also extensively studied as a potential biorenewable platform for the development of a plethora of well-promising, bioactive organic compounds [23,24,25,26,27,28,29]. However, it has never been explored as a chiral platform for the preparation of fluorinated, polycylic pyrazole derivatives.

The presented study showed that, in contrast to the widely known ‘classic’ *N*(Ph), *C*(Ph)-nitrile imine **9**, the (3+2)-cycloadditions of recently developed fluorinated nitrile imines **1**, derived from trifluoroacetonitrile, react with levoglucosenone **6** in a highly stereoselective manner, and the expected polycyclic pyrazolines **11** are formed in kinetically controlled reactions with perfect regioselectivity as *exo*-cycloadducts, exclusively. The DFT calculations fully support the experimental findings.

It was also established that the initially obtained pyrazolines can be smoothly oxidized to the corresponding pyrazoles by treatment with MnO_2_ in DMSO as a solvent. For all these reasons, the method described therein offers a new, convenient access to optically active, polycylic, trifluoromethyl substituted pyrazoles incorporating the chiral levoglucosenone skeleton, which can be of importance for their further medicinally and/or agrochemically-oriented studies.

This study emphasizes the universality and importance of 1,3-dipolar cycloaddition reactions for the current development of organic synthesis related to the preparation of five membrane nitrogen heterocycles [63,64,65].

## Data Availability

The data presented in this manuscript are available on request from the corresponding authors.

## Supplementary Materials

The following supporting information can be downloaded at: https://www.mdpi.com/article/10.3390/molecules28217348/s1. The authors have cited additional references within the Supporting Information [66,67,68,69,70].
