# Supplementary material for: (3+2)-Cycloadditions of Levoglucosenone (LGO) with Fluorinated Nitrile Imines Derived from Trifluoroacetonitrile: An Experimental and Computational Study
Source: Molecules. 2023 Oct 30;28(21):7348. doi: 10.3390/molecules28217348 (PMC10647924; doi:10.3390/molecules28217348)
Supplement: Supplementary file 1 [file molecules-28-07348-s001.zip › molecules-2680523-supplementary.pdf]

Supporting Materials

(3+2)-Cycloadditions of Levoglucosenone (LGO) with Fluorinated Nitrile Imines Derived from Trifluoroacetonitrile; An Experimental and Computational Study

Grzegorz Mlostoń,*[a] Katarzyna Urbaniak,[a] Marcin Palusiak,[b] Zbigniew Witczak,[c] Ernst-Ulrich Würthwein*[d]

[a] Department of Organic & Applied Chemistry, Faculty of Chemistry, University of Lodz, Tamka 12, PL-91-403 Lodz, Poland

[b] Department of Physical Chemistry, Faculty of Chemistry, University of Lodz, Pomorska 163/165, PL-90-236 Lodz, Poland

[c] Department of Pharmaceutical Sciences, Nesbitt School of Pharmacy, Wilkes University, 84 W. South Street, Wilkes-Barre, PA 18766, USA

[d] Organisch-Chemisches Institut and Center for Multiscale Theory and Computation (CMTC), Universität Münster, Corrensstrasse 40, D-48149 Münster, Germany

Contents:

Section 1: *General information* 2

Section 2: *(3+2)-Cycloadducts (fused pyrazolines)* ***11c-11h*** *and fused pyrazoles* ***12a-12b*** 3

Section 3: *^{1}H, ^{13}C NMR, and ^{19}F NMR spectra registered for (3+2)-cycloadducts (fused pyrazolines)* ***11a-11i*** *and for fused pyrazoles* ***12a-12c***. 12

Section 4: *X-Ray structure determination of fused pyrazole exo-**7** and pyrazoline **11a**.* 25

Section 5: *DFT calculations* 28

Section 6: *References* 39

Section 7. *List of captoons for schemes and figures of the main manuscript* 41

Section 1: *General information* (see also in Experimental)

1.1. Reagents and solvents

Reagents and solvents were, unless stated otherwise, used as commercially available with reagent grade and did not require further purification.

1.2. NMR spectroscopy: NMR spectra were recorded with a Bruker AVIII 600 (^{1}H NMR [600 MHz];^{13}C NMR [151 MHz]) or with a Varian Gemini 2000BB 200 MHz (^{19}F NMR [188 MHz]) instruments. Chemical shifts are reported relative to solvent residual peaks (^{1}H NMR, δ = 7.26 ppm [$CDCl_3$]; ^{13}C NMR, δ = 77.0 ppm [$CDCl_3$]) or to $CFCl_3$ (δ = 0.00 ppm) used as an external standard in ^{19}F NMR measurements.
In the ^{1}H NMR spectra, absorption signals of residual H_2O, hexane, CH_2Cl_2 and eventually also acetone are located at 1.60-1.62, 0.90-1.40, 5.30, and 2.00-2.10 ppm, respectively. The corresponding chemical shifts for these signals have been labeled in the registered spectra presented in the SI part.

1.3.FT-IR spectroscopy: IR spectra were run on the Agilent Cary 630 FTIR spectrometer.

1.4. Optical rotations were determined with an Anton Paar MCP 500 polarimeter at the temperatures indicated.

1.5. Flash chromatography: Products were purified by flash column chromatography (CC) on silica gel (230–400 mesh, Merck).

1.6. Preparative thin-layer chromatography (PLC) was carried out using 20x20 cm glass plates coated with silica (60 PF_{254}, Merck). In all cases separation of products was achieved using dichloromethane/ethanol (98:2) mixture.

1.7. Melting points were determined in capillaries with a MEL-TEMP apparatus (Aldrich) and are uncorrected.

1.7. Elemental analyses were obtained with a Vario EL III (Elementar Analysensysteme GmbH) instrument.

Section 2: *(3+2)-Cycloadducts (fused pyrazolines)* ***10/10'***, ***11c-11h***, *and fused pyrazoles 7/7'*, ***12a-12c***

Reaction of *C*(Ph),*N*(Ph)-nitrile imine (9) with levoglucosenone 6:

A portion of 126 mg (1 mmol) **6** and 285 mg (1.1 mmol) of the benzaldehyde hydrazonoil chloride were dissolved in 3 ml of dry THF, and an excess of NEt_3 (ca. 4 mmol) was added to the solution. The mixture was magnetically stirred over 24h. Then the solvent was evaporated, the crude mixtures were purified by chromatography (SiO_2 column) using petroleum ether with increasing amounts of methylene chloride as an eluent. The main fraction isolated as a yellowish, thick oil (205 mg, 58 %) was identified as a mixture of fused pyrazolines **10** and **10'**. All attempts to separate the mixture of isomeric products by standard methods (column chromatography, fractional crystallization) were unsuccessful and therefore both cycloadducts were characterized only by ^{1}H NMR spectroscopy registered for the isolated mixture.

1,3-Diphenyl-3a,4,5,8a-tetrahydro-1H-4,7-epoxyoxepino[4,5-c]pyrazol-8(7H)-one (*exo*-**10**, major) and *1,3-diphenyl-3a,7,8,8a-tetrahydro-1H-5,8-epoxyoxepino[4,5-c]pyrazol-4(5H)-one* (*exo*-**10'**, minor): yield: 230 mg (72%), isolated as yellowish oil, ratio of isomers ca. 65:35. ^{1}H NMR: δ (for *exo*-**10**) 4.20 (1H, d, $J_{H,H}$ = 11.9 Hz) and 5.03 (1H, $J_{H,H}$ = 11.9 Hz); (for *exo*-**10'**): ^{1}H NMR: δ 4.48 (1H, d, $J_{H,H}$ = 11.3 Hz) and 4.68 (1H, $J_{H,H}$ = 11.3 Hz).

Oxidation of isomeric pyrazolines 10 and 10' with MnO_2:

The isolated mixture of preliminarily purified pyrazolines **10**/**10'** (320 mg, 1 mmol) was dissolved in 20 mL DMSO and after addition of excess MnO_2 (8040 mg, 40 mmol), the magnetically stirred solution was heated on an oil bath over 15 h. Then the solution was filtered through celit and the collected black, solid material was washed with ethyl acetate. The combined organic fractions were diluted with water and shaken with three portions of ethyl acetate. The organic layers were separated and dried over $MgSO_4$. After filtration, the solvent was evaporated and the obtained oily material was purified by preparative layer chromatography. After four-times repeated development using dichloromethane as an eluent, the major component, i.e. pyrazole **7** was isolated as a less polar fraction and the minor product **7'** formed a slightly more polar fraction.

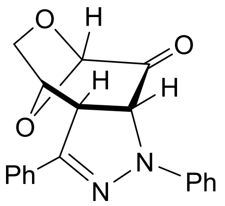

*1,3-Diphenyl-4,5-dihydro-1*H*-4,7-epoxyoxepino[4,5-*c*]pyrazol-8(7*H*)-one* (**7**). Yield: 90 mg (28 %), colorless crystals, m.p. 165–166 °C (petroleum ether/CH_2Cl_2).

^{1}H NMR ($CDCl_3$): δ 4.03 (*d*, $J_{H,H}$, = 6.5 Hz, 1CH); 4.21 (*dd*, $J_{H,H}$, = 6.5 Hz, $J_{H,H}$, = 4.3 Hz, 1CH); 5.57 (*s*, 1CH); 5.88 (*d*, $J_{H,H}$, = 4.2 Hz, 1CH); 7.46-7.53 (*m*, $6CH_{arom}$); 7.69-7.73 (*m*, $4CH_{arom}$);

^{13}C NMR (CDCl;$_3$): δ 68.1 (CH_2); 71.6, 101.2 (2CH); 124.3, 127.3, 128.7, 128.8, 128.9, 129.1 ($10CH_{arom}$); 128.3, 131.5, 132.4, 138.9, 146.9 (2C=, $2C_{arom}$, C=N); 179.8 (C=O);

IR: ν 1695*s* (C=O), 1603*m*, 1505*s*, 1438*s*, 1326*m*, 1233*m*, 1185*m*, 1095*m*, 995*s*, 894*s*, 743*vs*, cm^{-1}.

EA for $C_{19}H_{14}N_2O_3$ (318.10): calcd. C 71.69; H 4.43: N 8.80; found C 71.73; H 4.26: N 8.57.

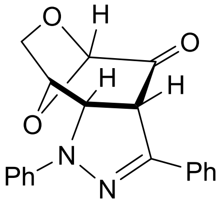

*1,3-Diphenyl-7,8-dihydro-1*H*-5,8-epoxyoxepino[4,5-*c*]pyrazol-4(5*H*)-one* (**7'**). Yield: 25 mg (8 %), colorless crystals, m.p. 147–149 °C (petroleum ether/CH_2Cl_2).

^{1}H NMR ($CDCl_3$): δ 4.11 (*d*, $J_{H,H}$, = 6.9 Hz, 1CH); 4.20 (*dd*, $J_{H,H}$, = 6.9 Hz, $J_{H,H}$, = 4.5 Hz, 1CH); 5.59 (*s*, 1CH); 5.68 (*d*, $J_{H,H}$, = 4.4 Hz, 1CH); 7.46-7.53 (*m*, $8CH_{arom}$); 8.29-8.39 (*m*, $2CH_{arom}$);

IR: ν 1700 *s* (C=O), 1591*s* 1494*s*, 1436*m*, 1364*m*, 1267*m*, 1165*m*, 1099*m*, 972*s*, 834*m*, 768*vs*, cm^{-1}.

EA for $C_{19}H_{14}N_2O_3$ (318.10): calcd. C 71.69; H 4.43: N 8.80; found C 71.67; H 4.37: N 8.89.

(3+2)-Cycloadditions of nitrile imine 1c-1i with levoglucosenone 6: for general procedure, see: Experimental.

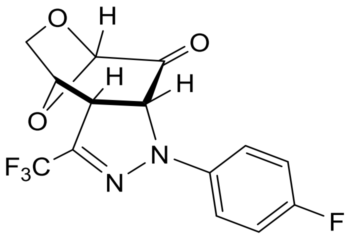

5-(4-Fluorophenyl)-3-(trifluoromethyl)-9,11-dioxa-4,5-diazatricyclo[6.2.1.02,6]undec-3-en-7-one (*exo*-**11c**). Yield:155 mg (47 %), pale yellow crystals, mp = 89–91 ºC (petroleum ether/CH_2Cl_2).

^{1}H NMR ($CDCl_3$): δ 4.02-4.04 (*m*, 1H); 4.08-4.10 (*m*, 1H); 4.12-4.14 (*m*, 1H); 5.15 (*d*, $J_{H,H}$ = 4.7 Hz, 1H); 5.23 (*d*, $J_{H,H}$ = 12.0 Hz, 1H); 5.29 (*d*, $J_{H,H}$ = 11.4 Hz, 1H); 7.01-7.04 (*m*, 2CH_{arom}); 7.14-7.17 (*m*, 2CH_{arom});

^{13}C NMR ($CDCl_3$): δ 52.0, 63.7, 71.0, 100.10, 68.6, 115.7, 115.8, 115.9, 116.0, 120.5 (*q*, $^1J_{C,F}$ = 288.2 Hz, *C*F_3); 134.8 (*q*, $^2J_{C,F}$ = 37.4 Hz, *C*-CF_3), 139.3 (N-C_{arom}); 158.3 (*d*, $^1J_{C,F}$ = 239.4 Hz, F-C_{arom}); 142.0, 194.3 (C=O).

^{19}F ($CDCl_3$): δ –63.9 (C*F*$_3$), –122.2 (*F*C_{ar});

IR: ν 1752*s* (C=O), 1590*m*, 1509*s*, 1423*m*, 1312*s*, 1238*m*, 1185*m*, 1133*s*, 1088*vs*, 1028*m*, 973*s*, 917*s*, 827*s*, 724*m*, cm^{-1};

EA for $C_{14}H_{10}F_4N_2O_3$ (330.24): calcd. C 50.92, H 3.05, N 8.48; found C 50.65, H 3.16, N 8.59.

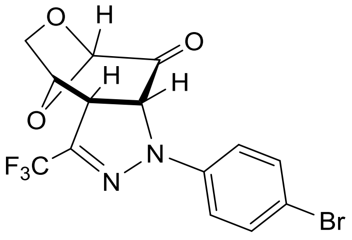

5-(4-Bromophenyl)-3-(trifluoromethyl)-9,11-dioxa-4,5-diazatricyclo[6.2.1.02,6]undec-3-en-7-one (*exo*-**11d**). Yield: 205 mg (52 %), pale yellow crystals, mp = 100–101 ºC (petroleum ether/CH_2Cl_2). $[\alpha]_D^{20}$ = –706 (*c* 0.3, $CHCl_3$).

^{1}H NMR ($CDCl_3$): δ 3.99-4.01 (*m*, 1H); 4.06-4.08 (*m*, 1H); 4.10-4.12 (*m*, 1H); 5.14 (*d*, $J_{H,H}$ = 4.6 Hz, 1H); 5.22 (*d*, $J_{H,H}$ = 12.0 Hz, 1H); 5.27 (*d*, $J_{H,H}$ = 10.9 Hz, 1H); 7.05, 7.41 (AB-system, $J_{H,H}$ = 8.9 Hz, 4CHarom);

^{13}C NMR ($CDCl_3$): δ 52.1, 63.3, 70.8, 100.1, 68.6, 116.0, 132.1, 120.8 (*q*, $^1J_{C,F}$ = 268.2 Hz, C*F*$_3$); 135.9 (*q*, $^2J_{C,F}$ = 37.3 Hz, *C*-CF_3); 114.3, 142.0, 194.3 (C=O);

^{19}F ($CDCl_3$): δ –64.0;

IR: ν 1751*s* (C=O), 1587*s*, 1407*m*, 1315*s*, 1258*m*, 1181*s*, 1092*vs*, 1082*vs*, 976*s*, 916*s*, 827*s*, 721*m*, cm^{-1};

EA for $C_{14}H_{10}BrF_3N_2O_3$ (391.14): calcd. C 42.99, H 2.58, N 7.16; found C 43.17, H 2.86, N 7.33.

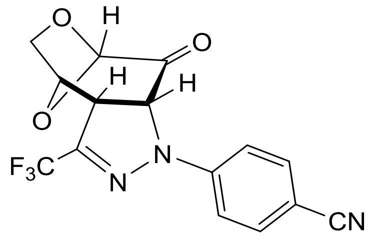

4-[7-Oxo-3-(trifluoromethyl)-9,11-dioxa-4,5-diazatricyclo[6.2.1.02,6]undec-3-en-5-yl]benzonitrile (*exo*-**11e**). Yield: 180 mg (53 %), pale yellow crystals, mp = 170–171 °C (petroleum ether/CH_2Cl_2). $[\alpha]_D^{20}$ = –676 (*c* 0.3, $CHCl_3$).

^1H NMR ($CDCl_3$): δ 4.00-4.08 (*m*, 1H); 4.10-4.13 (*m*, 1H); 4.16-4.18 (*m*, 1H); 5.18 (*d*, $J_{H,H}$ = 4.7 Hz, 1H); 5.30 (*brs*, 1H); 5.35 (*d*, $J_{H,H}$ = 11.6 Hz, 1H); 7.22, 7.57 (*AB*-system, $J_{H,H}$ = 8.9 Hz, 4CHarom);

^{13}C NMR ($CDCl_3$): δ 52.3, 62.8, 70.7, 100.1, 68.6, 114.4, 133.4, 120.3 (*q*, $^1J_{C,F}$ = 269.0 Hz, C-C*F*3); 137.7 (*q*, $^2J_{C,F}$ = 38.0 Hz, *C*-CF_3); 119.3, 146.0, 193.6 (C=O);

^{19}F ($CDCl_3$): δ –64.4;

IR: ν 2238*s* (CN); 1744*s* (C=O), 1599*s*, 1423*m*, 1319*s*, 1267*m*, 1177*s*, 1121*vs*, 1092*vs*, 969*s*, 910*s*, 827*s*, 730*m*, cm^{-1}.

EA for $C_{15}H_{10}F_3N_3O_3$ (326.28): calcd. C 53.42, H 2.99, N 12.46; found C 53.47, H 3.00, N 12.59.

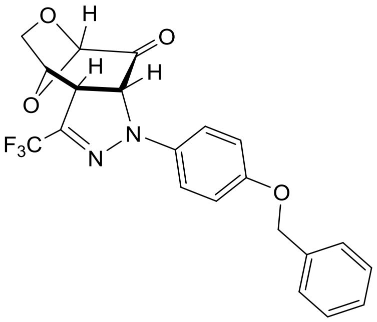

5-(4-Benzyloxyphenyl)-3-(trifluoromethyl)-9,11-dioxa-4,5-diazatricyclo[6.2.1.02,6]undec-3-en-7-one (*exo*-**11f**). Yield: 230 mg (55 %), pale yellow crystals, mp = 118–119 °C (petroleum ether/CH_2Cl_2). $[\alpha]_D^{20}$ = –909 (*c* 0.3, $CHCl_3$).

^{1}H NMR ($CDCl_3$): δ 3.99 (*d*, $J_{H,H}$ = 12.3 Hz, 1H); 4.06-4.08 (*m*, 1H); 4.10-4.11 (*m*, 1H); 5.06 (*s*, H_2C-Ph); 5.12-5.16 (*m*, 1H); 5.17-5.20 (*m*, 1H); 6.96, 7.14 (*AB*-system, $J_{H,H}$ = 9.0 Hz, 4CH_{arom}); 7.33-7.37 (*m*, 1CH_{arom}); 7.39-7.43 (*m*, 2CH_{arom}); 7.44-7.47 (*m*, 2CH_{arom});

^{13}C NMR ($CDCl_3$): δ 51.8, 64.0, 68.6, 70.5, 71.0, 100.1, 115.6, 116.0, 127.5, 128.6, 120.9 (*q*, $^1J_{C,F}$ = 267.8.0 Hz, C-*CF*$_3$); 133.8 (*q*, $^2J_{C,F}$ = 37.2 Hz, *C*-CF_3); 128.0, 137.1, 154.3, 194.5 (C=O);

^{19}F NMR ($CDCl_3$): δ –63.8;

IR: ν 1753*s* (C=O), 1589*s*, 1453*m*, 1411*m*, 1308*s*, 1244*s*, 1174*s*, 1095*vs*, 1047*s*, 965*s*, 916*s*, 820*s*, 745*s*, cm^{-1};

EA for $C_{21}H_{17}F_3N_2O_3$ (418.37): calcd. C 60.29, H 4.10, N 6.70; found C 60.25, H 4.10, N 6.85.

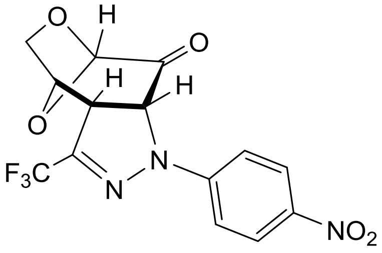

5-(4-Nitrophenyl)-3-(trifluoromethyl)-9,11-dioxa-4,5-diazatricyclo[6.2.1.02,6]undec-3-en-7-one (*exo*-**11g**). Yield: 315 mg (88 %), yellow crystals, mp = 91–92 °C (petroleum ether/CH_2Cl_2). $[\alpha]_D^{20}$ = –751 (*c* 0.2, $CHCl_3$).

^{1}H NMR ($CDCl_3$): δ 4.01-4.04 (*m*, 1H); 4.09-4.11 (*m*, 1H); 4.11 (*d*, $J_{H,H}$ = 8.9 Hz, 1H); 5.11 (*d*, $J_{H,H}$ = 4.7 Hz, 1H); 5.22 (*brs*, 1H); 5.30 (*d*, $J_{H,H}$ = 11.5 Hz, 1H); 7.12, 8.09 (*A*B-system, $J_{H,H}$ = 9.3 Hz 4CHarom);

^{13}C NMR ($CDCl_3$): δ 52.4, 62.9, 68.7, 100.1, 70.6, 113.8, 125.5, 120.2 (*q*, $^{1}J_{C,F}$ = 278.4 Hz, C-*CF*$_3$); 138.9 (*q*, $^{2}J_{C,F}$ = 37.9 Hz, *C*-CF_3); 141.8, 147.7, 193.3 (C=O);

^{19}F ($CDCl_3$): δ –64.5;

IR: ν 1751*s* (C=O), 1587*m*, 1502*s*, 1426*m*, 1304*s*, 1226*m*, 1181*m*, 1095*vs*, 1027*m*, 972*s*, 915*m*, 837*s*, 749*m*, cm^{-1};

EA for $C_{14}H_{10}F_3N_3O_5$ (357.25): calcd. C 47.07, H 2.82, N 11.76; found C 47.08, H 2.72, N 11.70.

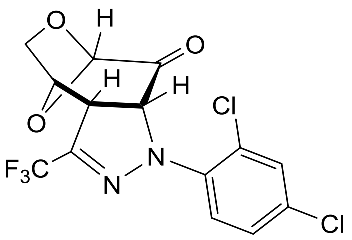

5-(2,4-Dichlorophenyl)-3-(trifluoromethyl)-9,11-dioxa-4,5-diazatricyclo[6.2.1.02,6]undeca-3-en-7-one (*exo*-**11h**). Yield: 190 mg (50%), colorless crystals, mp = 157–159 °C (petroleum ether/CH_2Cl_2). $[\alpha]_D^{20}$ = –1286 (*c* 0.3, $CHCl_3$).

^{1}H NMR ($CDCl_3$): δ 3.92-3.98 (2CH); 4.06 (*d*, $J_{H,H}$ = 7.8 Hz, 1CH); 5.03 (*s*,1CH); 5.07 (*d*, $J_{H,H}$ = 4.8 Hz, 1CH); 5.61 (*d*, $J_{H,H}$ = 11.3 Hz, 1CH); 7.15-7.18 (*m*, $1CH_{arom}$); 7.30-7.32 (*m*, $2CH_{arom}$);

^{13}C NMR ($CDCl_3$): δ 52.0, 65.2, 70.8, 99.9, 68.2, 120.7 (*q*, $^{1}J_{C,F}$ = 269.7 Hz, C-*CF*$_3$); 125.2, 127.8, 129.7, 124.9, 130.8, 139.9, 137.0 (*q*, $J_{H,H}$ = 37.1 Hz, *C*-CF_3); 194.4 (C=O);

^{19}F: δ –64.1;

IR: ν 1744*s* (C=O), 1610*m*, 1479*s*, 1379*m*, 1300*m*, 1259*m*, 1181*s*, 1103*vs*, 1017*s*, 969*s*, 872*s*, 823*s*, 699*s*, cm^{-1};

EA for $C_{14}H_9Cl_2F_3N_2O_3$ (381.13): calcd. C 44.12, H 2.38, N 7.35; found C 43.92, H 2.44, N 7.32.

Reactions of nitrile imine 1i with levoglucosenone 6:

The reaction was performed following general procedure described for **1a**-**1h**.

In the ^{1}H NMR spectrum of the crude reaction mixture obtained from **1i** the major product was identified as the expected pyrazoline *exo*-**11i**. After column chromatography (SiO_2, PE/CH_2Cl_2) the pyrazole **12a** was isolated as a sole product.

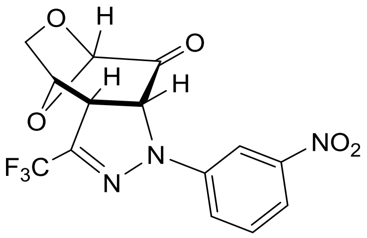

5-(3-Nitrophenyl)-3-(trifluoromethyl)-9,11-dioxa-4,5-diazatricyclo[6.2.1.02,6]undec-3-en-7-one (*exo*-**11i**) (identified by ^{1}H NMR in the spectrum registered for crude reaction mixture).

^{1}H NMR: δ 3.98-4.04 (2CH); 4.09 (*d*, $J_{H,H}$ = 8.0 Hz, 1CH); 5.10 (*d*, $J_{H,H}$ = 4.7 Hz, 1CH); 5.21 (*d*, $J_{H,H}$ = 7.6 Hz, 1CH); 5.28 (*d*, $J_{H,H}$ = 11.7 Hz, 1CH); 7.37 (*t*, $J_{H,H}$ = 8.2 Hz, 1CH$_{arom}$); 7.43-7.46 (*m*, 1CH$_{arom}$); 7.74-7.76 (*m*, 1CH$_{arom}$); 7.87-7.89 (*m*, 1CH$_{arom}$).

5-(3-Nitrophenyl)-3-(trifluoromethyl)-9,11-dioxa-4,5-diazatricyclo[6.2.1.02,6]undeca-2(6),3-dien-7-one (**12a**). Yield: 150 mg (42 %), yellowish, viscous oil. $[\alpha]_D^{20}$ = –1811 (*c* 0.2, $CHCl_3$).

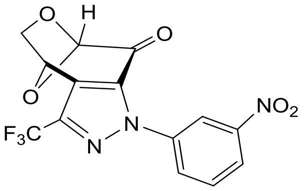

^{1}H NMR ($CDCl_3$): δ 3.91 (*d*, $J_{H,H}$ = 7.1 Hz, 1CH); 4.16 (*dd*, $J_{H,H}$ = 7.1 Hz, $J_{H,H}$ = 4.3 Hz, 1CH$_{arom}$); 5.61 (*s*, 1CH); 5.81 (*d*, $J_{H,H}$ = 4.1 Hz, 1CH); 7.45 (*t*, $J_{H,H}$ = 8.2 Hz, 1CH$_{arom}$); 8.09-8.09 (*m*, 1CH$_{arom}$); 8.36-8.38 (*m*, 1CH$_{arom}$); 8.63-8.64 (*m*, 1CH$_{arom}$);

^{13}C NMR ($CDCl_3$): δ 68.0 (CH_2), 70.5, 100.8, 120.5 (*q*, $^1J_{C,F}$ = 260.7 Hz, C-*CF*$_3$); 119.7, 124.2, 129.9, 130.0, 129.4, 133.2, 138.2 (*q*, $^2J_{C,F}$ = 39.8 Hz, *C*-CF$_3$), 138.7, 148.4 (O_2N-*C*$_{ar}$), 178.8 (C=O);

^{19}F NMR ($CDCl_3$): δ –61.0;

IR: ν 1722s (C=O), 1531s, 1379m, 1349s, 1252s, 1200m, 1133s, 1077vs, 1921m, 961m, 902s, 849s, 738s, 678s, cm^{-1}.

EA for $C_{14}H_8F_3N_3O_5$ (355.23): calcd. C 47.34, H 2.27, N 11.83; found C 47.49, H 2.47, N 11.54.

Oxidation of fluorinated pyrazoline *exo*-11g with MnO_2:

The reaction was performed based on analogous procedure as described for the mixture **10/10’** (see above). The crude product was purified by column chromatography (SiO_2, CH_2Cl_2) and analyzed as a viscous oil.

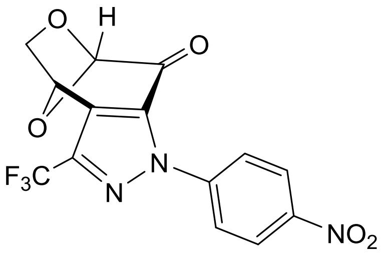

1-(4-Nitrophenyl)-3-(trifluoromethyl)-4,5-dihydro-1H-4,7-epoxyoxepino[4,5-c]pyrazol-8(7H)-one (**12b**). Yield: 185 mg (53%), yellowish, viscous oil.

^{1}H NMR ($CDCl_3$): δ 3.91 (*d*, $J_{H,H}$ = 7.14 Hz, 1CH); 4.16 (*dd*, $J_{H,H}$ = 7.14 Hz, $J_{H,H}$ = 2.13 Hz 1CH); 5.61 (s, 1CH); 5.80 (*d*, $J_{H,H}$ = 4.08 Hz, 1CH); 7.97, 8.41 (AB-system, $J_{H,H}$ = 9.06 Hz, 4CH_{arom});

^{13}C NMR ($CDCl_3$): δ 68.0, 70.5, 100.8, 124.6, 124.9, 120.4 (*q*, $^{1}J_{C,F}$ = 268.2 Hz, C-*CF*$_3$); 129.8, 133.47, 142.45, 147.9 138.5 (*q*, $^{2}J_{C,F}$ = 39.9 Hz, *C*-CF_3); 178.8 (C=O);

^{19}F ($CDCl_3$): δ –61.1.

IR: ν 1722*s* (C=O), 1531*s*, 1498*s*, 1338*m*, 1252*m*, 1110*s*, 1077*s*, 1008*m*, 902*s*, 857*s*, 723*vs*, cm^{1};

EA for $C_{14}H_8F_3N_3O_5$ (355.23): calcd. C 47.34, H 2.27, N 11.83; found C 47.25, H 2.51, N 11.69.

Attempted oxidation of pyrazolines *exo*-11g with trichloroisocyanuric acid (TCCA):

To magnetically stirred solution of *exo*-**11g** (357 mg, 1 mmol) in 3 mL CH_3CN, TCCA (174 mg, 0.75 mmol) was added in small portions . Progress of the reaction was monitored by TLC and after 15 min. no starting *exo*-**11g** was found in the mixture. The solvent was evaporated and crude products were isolated by column chromatography (SiO_2, CH_2Cl_2) as a mixture (ratio, ca. 85:15). All attempts to separate **12b** and **13c** by chromatography or by fractional crystallization were unsuccessful. Therefore, the unknown pyrazole **12c** was characterized by the ^{1}H and ^{13}C NMR spectra as a minor component in the mixture with **12b**.

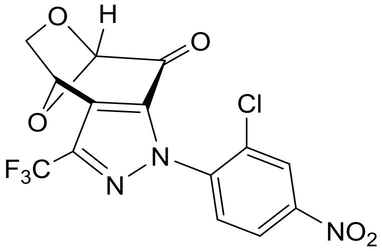

1-(2-Chloro-4-nitrophenyl)-3-(trifluoromethyl)-4,5-dihydro-1H-4,7-epoxyoxepino[4,5-c]pyrazol-8(7H)-one (**12c**); identified in the mixture with **12b** after column chromatography (SiO_2, CH_2Cl_2).

^{1}H NMR ($CDCl_3$): δ 3.93 (*d*, $J_{H,H}$ = 7.14 Hz, 1CH); 4.16-4.18 (*m*, 1CH); 5.56 (s, 1CH); 5.80-5.81 (*m*, 1CH); 7.70 (*d*, $J_{H,H}$ = 8.70 Hz, $1CH_{arom}$); 8.31 (*dd*, $J_{H,H}$ = 8.70 Hz, $J_{H,H}$ = 2.45 Hz, $1CH_{arom}$); 8.47 (*d*, $J_{H,H}$ = 2.45 Hz, $1CH_{arom}$);

^{13}C NMR ($CDCl_3$): δ 68.1, 70.6, 100.4, 122.7, 125.8, 129.5, 120.7 (*q*, $^1J_{C,F}$ = 268.6 Hz, C-*CF*$_3$); 128.1, 132.9, 135.0, 140.7, 149.0, 138.6 (*q*, $^2J_{H,H}$ = 39.9 Hz, C-CF_3); 178.5 (C=O).

Attempted air oxidation of pyrazoline *exo*-11g:

The solution of *exo*-**11g** (178 mg, 0.5 mmol) in 3 ml of dry toluene was heated under reflux condenser for 1.5h. After this time toluene was evaporated and the oily residue was analyzed by running the ^{1}H NMR spectrum. The 80:20 ratio of unconverted *exo*-**11g** and the expected oxdidation product **12b** was established by comparison of intensities of integration lines of characteristic signals located at 5.11 (*d*, 1H) and 5.80 (*d*, 1H) ppm, respectively.

Section 3: *¹H, ¹³C, and ¹⁹F NMR spectra registered for (3+2)-cycloadducts (fused pyrazolines)* ***11a-11h*** *and for fused pyrazoles* ***12a-12b***

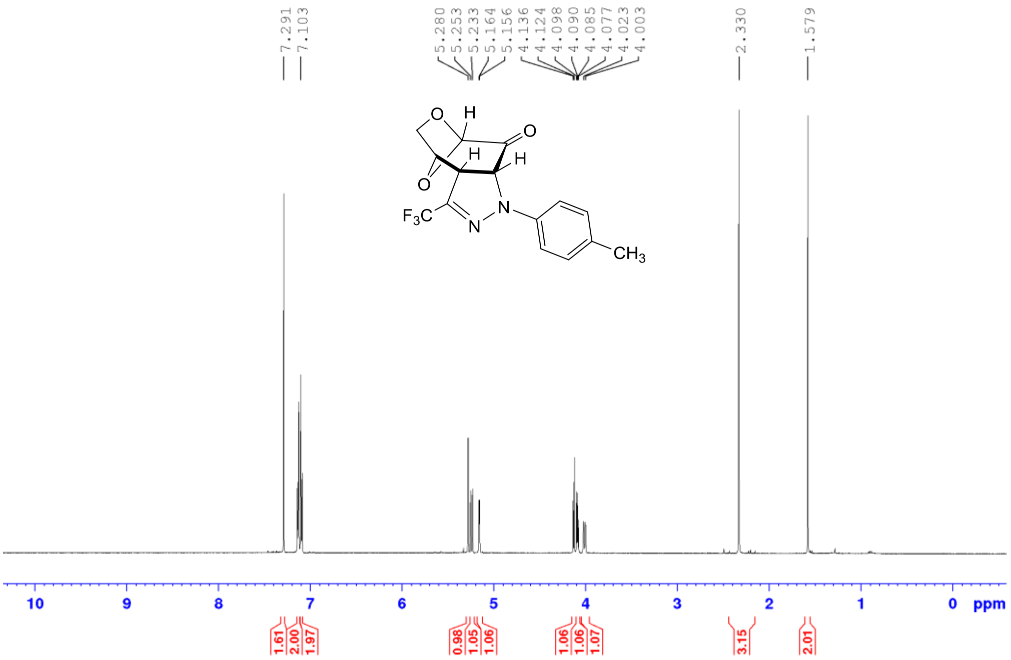

Fig. 1a. The ^{1}H NMR spectrum of (3+2)-cycloadduct **11a**.

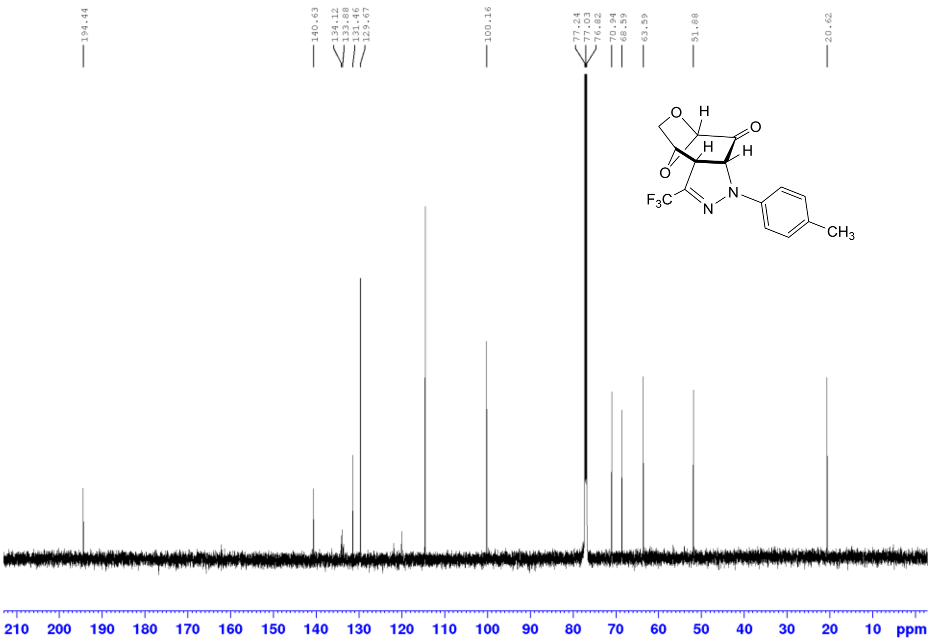

Fig. 1b. The ^{13}C NMR spectrum of (3+2)-cycloadduct *exo*-**11a**.

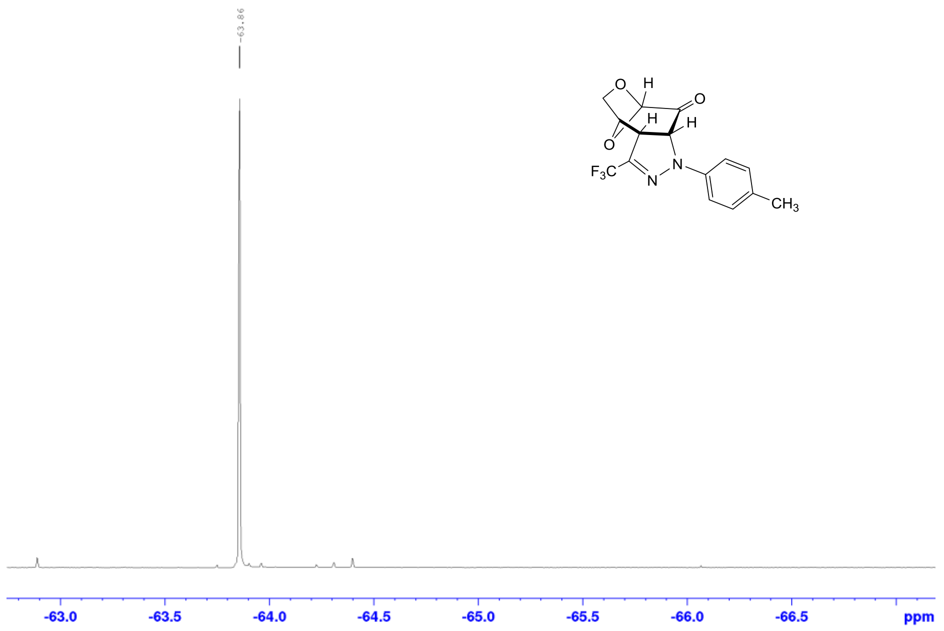

Fig. 1c. The ^{19}F NMR spectrum of (3+2)-cycloadduct *exo*-**11a**.

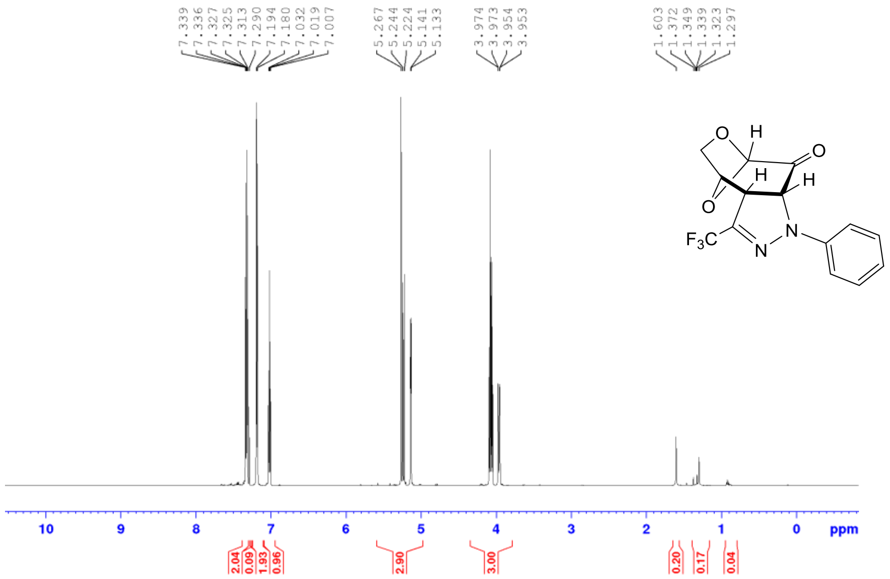

Fig. 2a. The ^{1}H NMR spectrum of (3+2)-cycloadduct *exo*-**11b**.

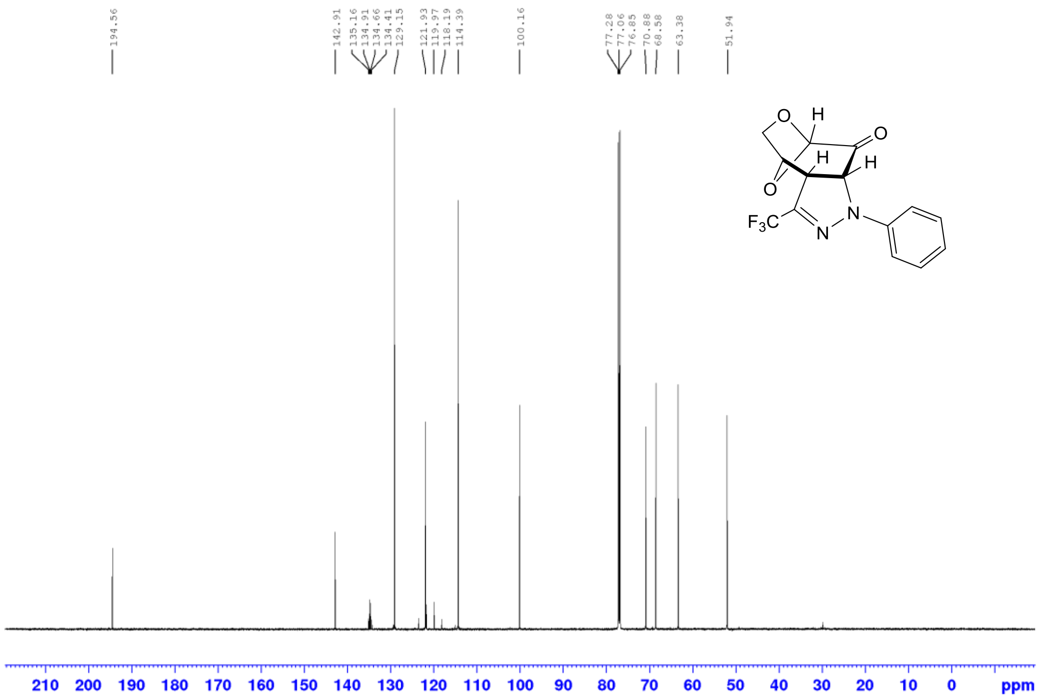

Fig. 2b. The ^{13}C NMR spectrum of (3+2)-cycloadduct *exo*-**11b**.

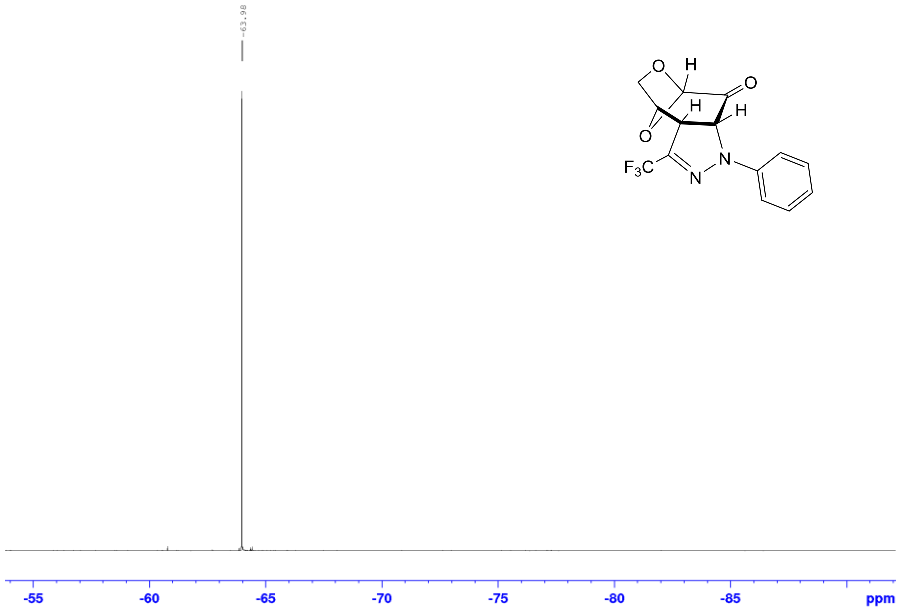

Fig. 2c. The ^{19}F NMR spectrum of (3+2)-cycloadduct *exo*-**11b**.

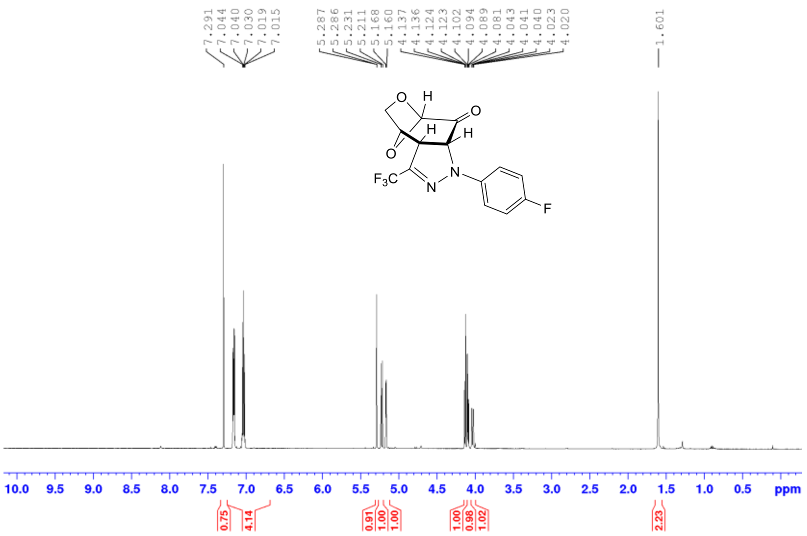

Fig. 3a. The ^{1}H NMR spectrum of (3+2)-cycloadduct *exo*-**11c**.

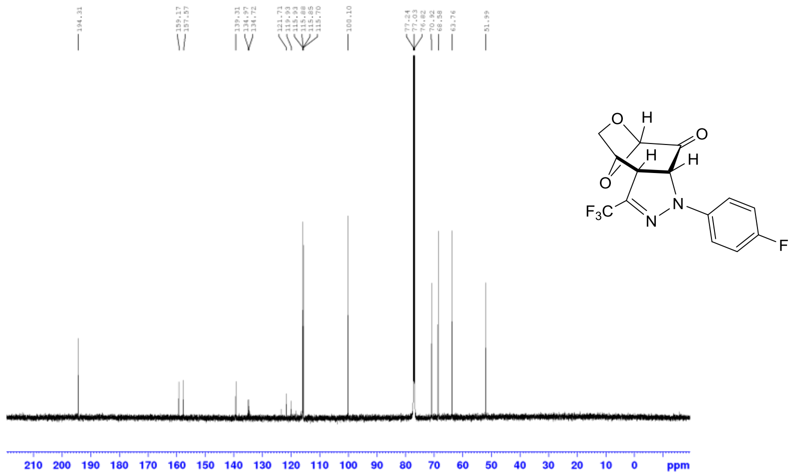

Fig. 3b. The ^{13}C NMR spectrum of (3+2)-cycloadduct *exo*-**11c**.

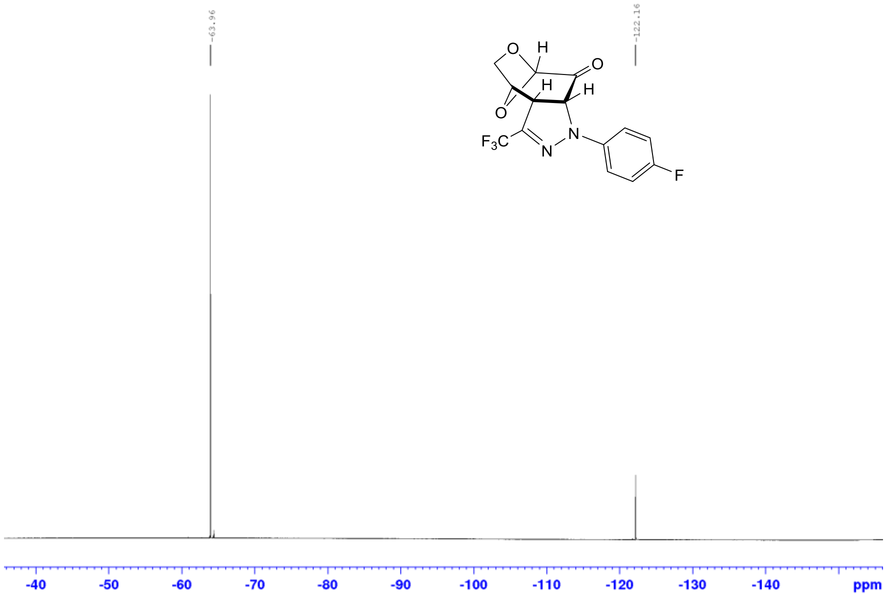

Fig. 3c. The ^{19}F NMR spectrum of (3+2)-cycloadduct *exo*-**11c**.

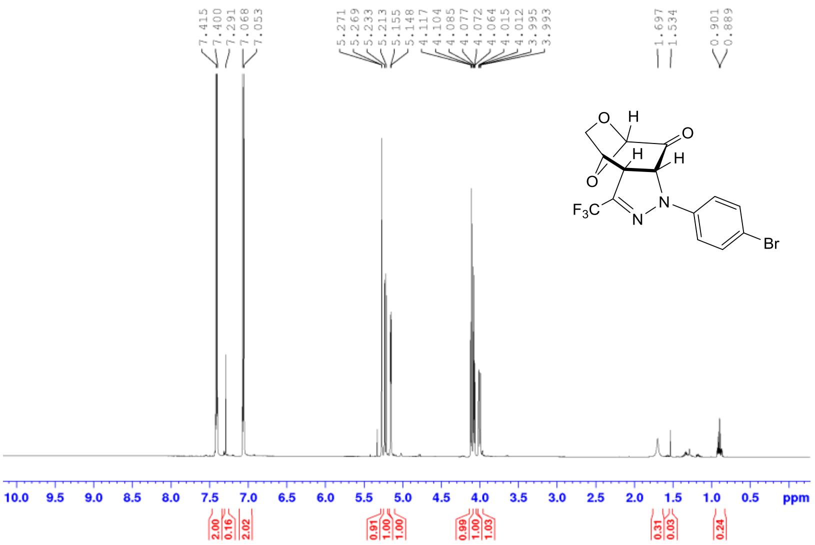

Fig. 4a. The ^{1}H NMR spectrum of (3+2)-cycloadduct *exo*-**11d**.

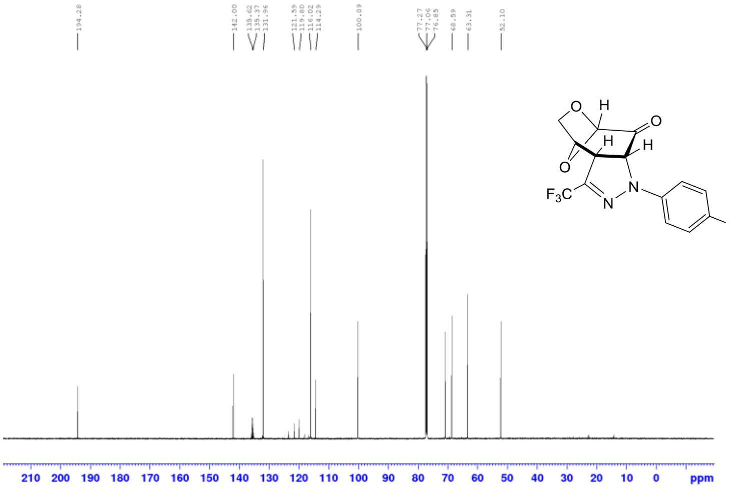

Fig. 4b. The ^{13}C NMR spectrum of (3+2)-cycloadduct *exo*-**11d**.

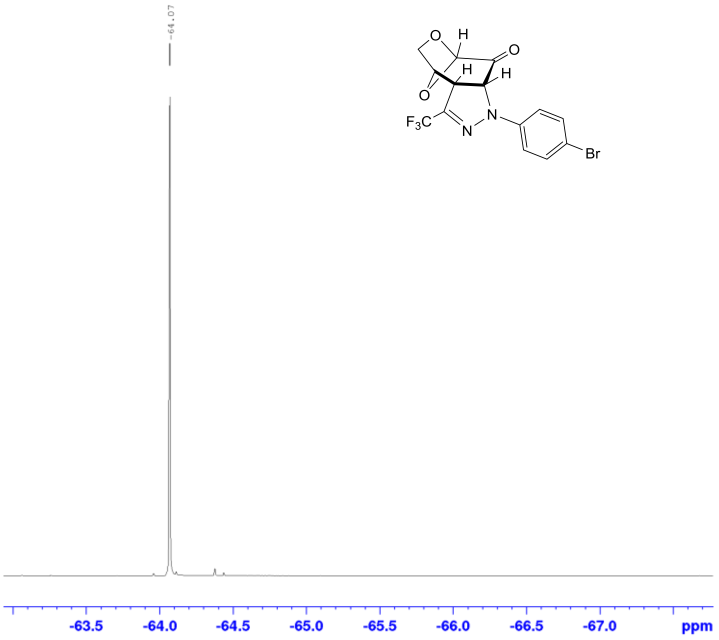

Fig. 4c. The ^{19}F NMR spectrum of (3+2)-cycloadduct *exo*-**11d**.

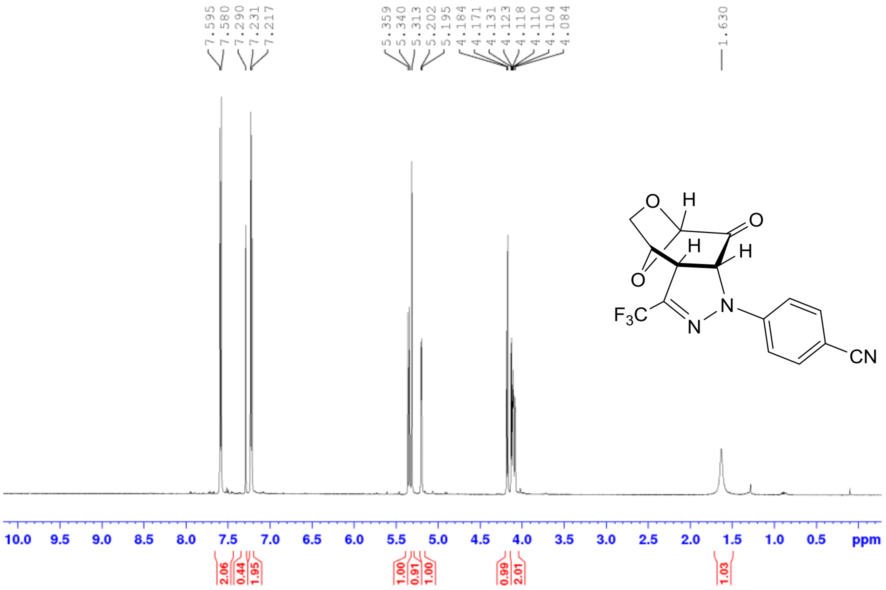

Fig. 5a. The ^{1}H NMR spectrum of (3+2)-cycloadduct *exo*-**11e**.

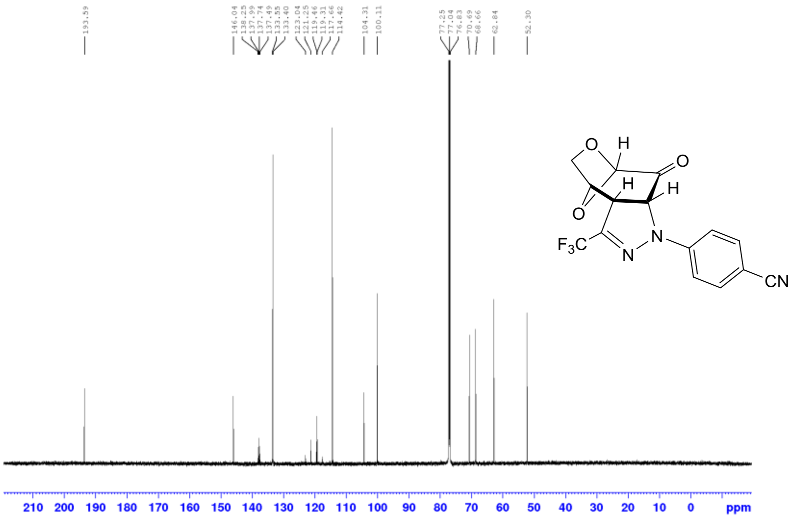

Fig. 5b. The ^{13}C NMR spectrum of (3+2)-cycloadduct *exo*-**11e**.

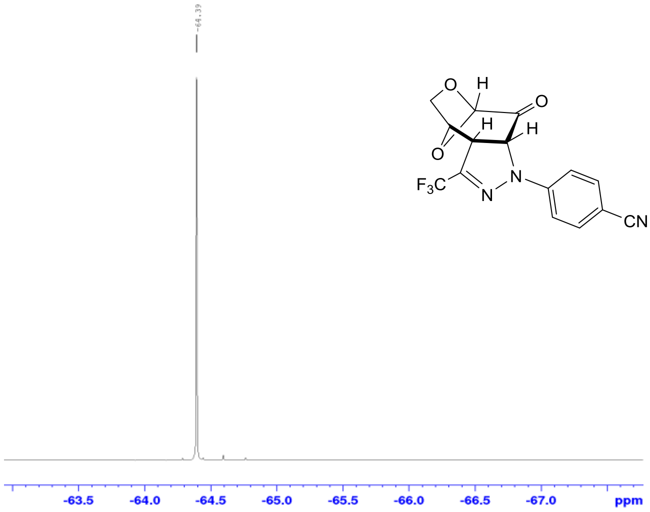

Fig. 5c. The ^{19}F NMR spectrum of (3+2)-cycloadduct *exo*-**11e**.

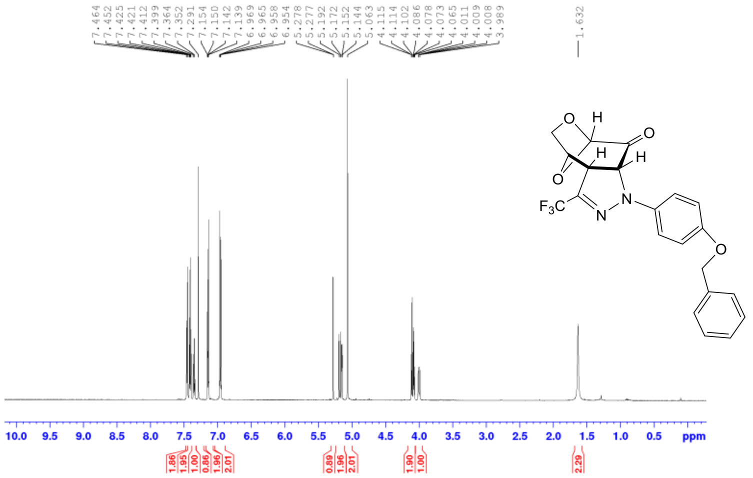

Fig. 6a. The ^{1}H NMR spectrum of (3+2)-cycloadduct *exo*-**11f**.

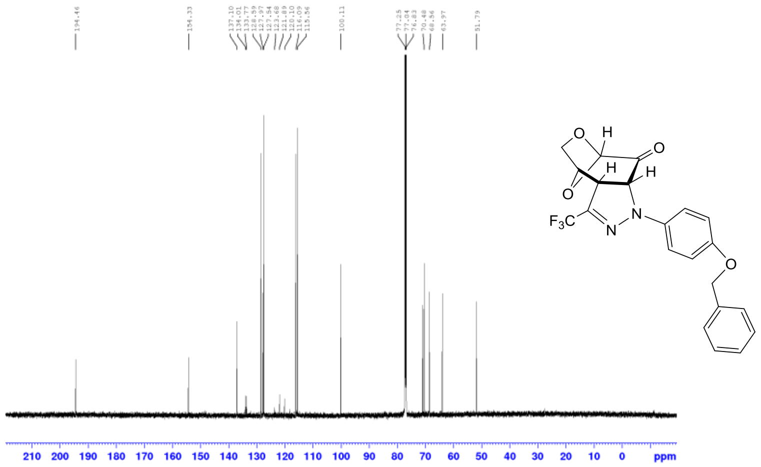

Fig. 6b. The ^{13}C NMR spectrum of (3+2)-cycloadduct *exo*-**11f**.

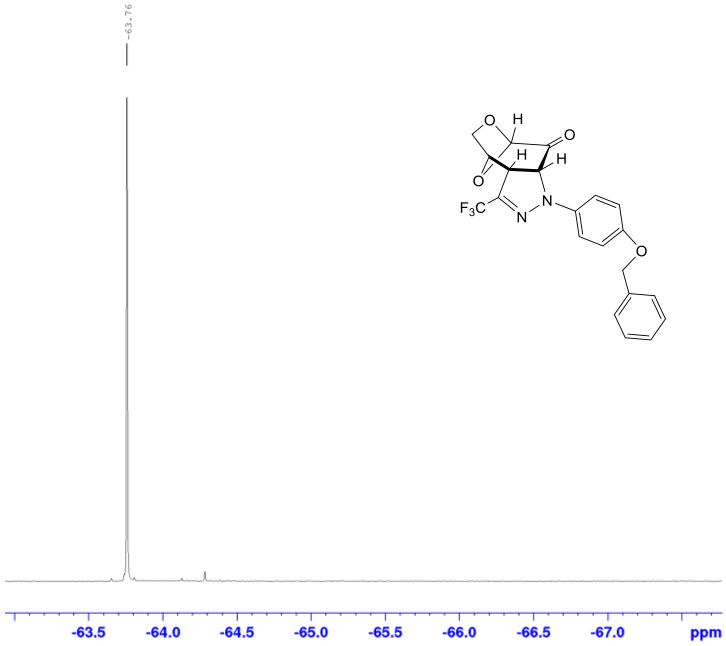

Fig. 6c. The ^{19}F NMR spectrum of (3+2)-cycloadduct *exo*-**11f**.

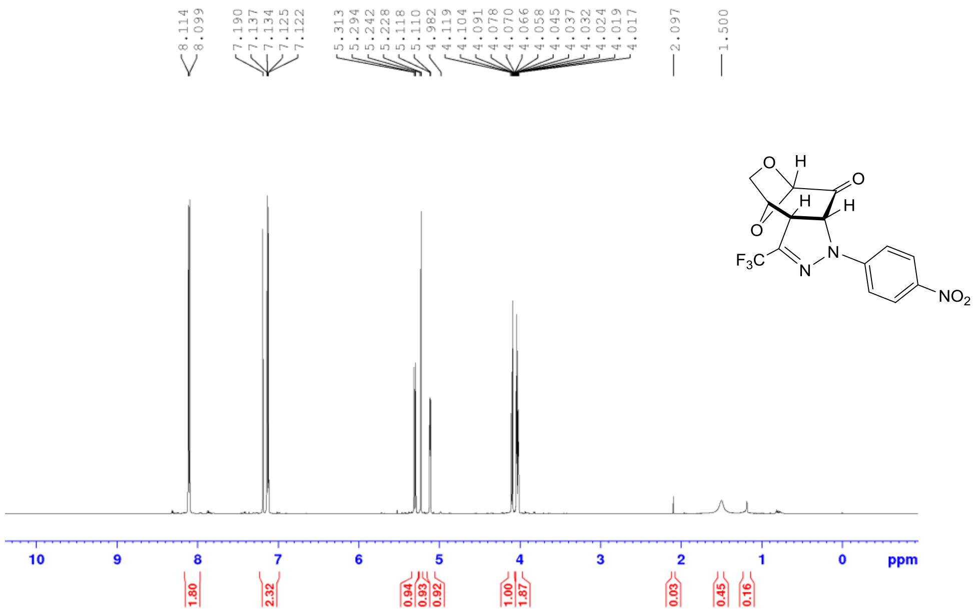

Fig. 7a. The ^{1}H NMR spectrum of (3+2)-cycloadduct *exo*-**11g**.

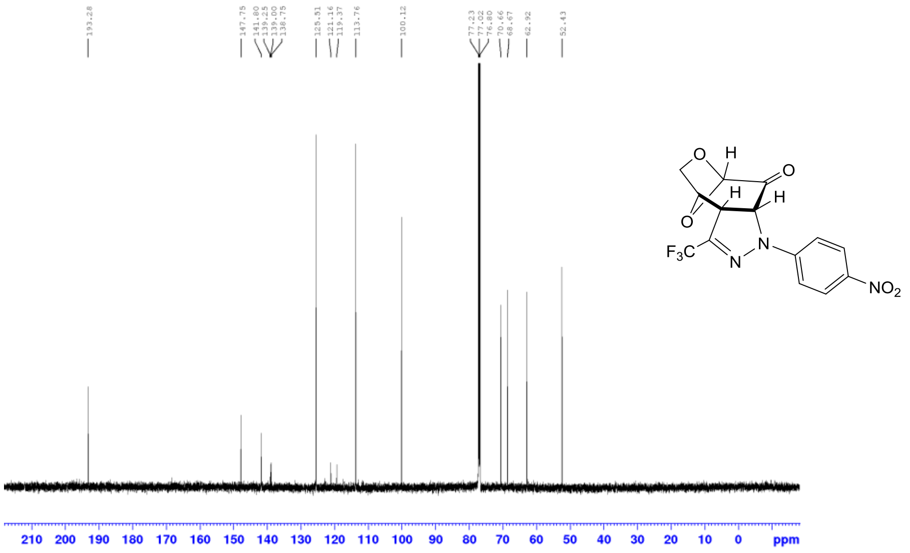

Fig. 7b. The ^{13}C NMR spectrum of (3+2)-cycloadduct *exo*-**11g**.

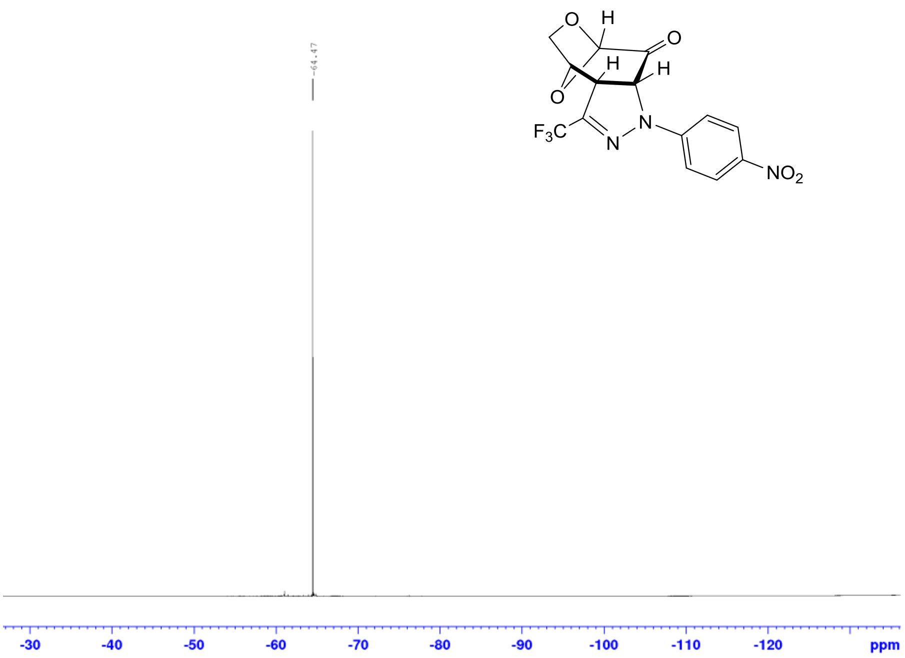

Fig. 7c. The ^{19}F NMR spectrum of (3+2)-cycloadduct *exo*-**11g**.

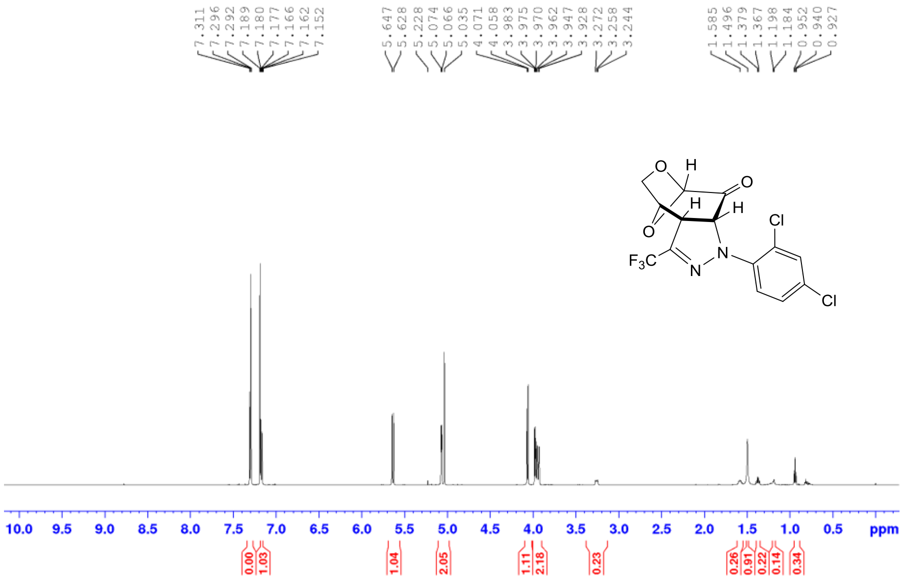

Fig. 8a. The ^{1}H NMR spectrum of (3+2)-cycloadduct *exo*-**11h**.

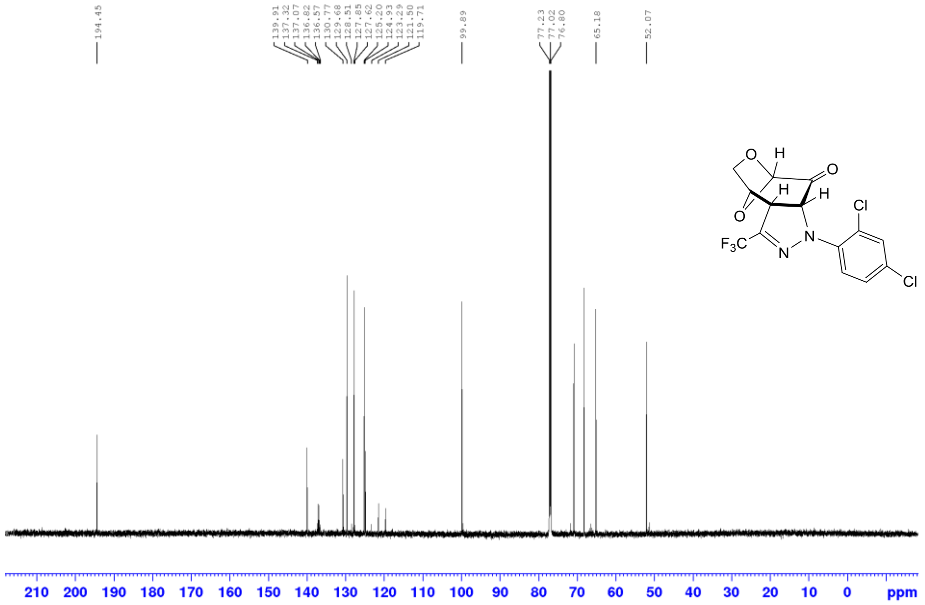

Fig. 8b. The ^{13}C NMR spectrum of (3+2)-cycloadduct *exo*-**11h**.

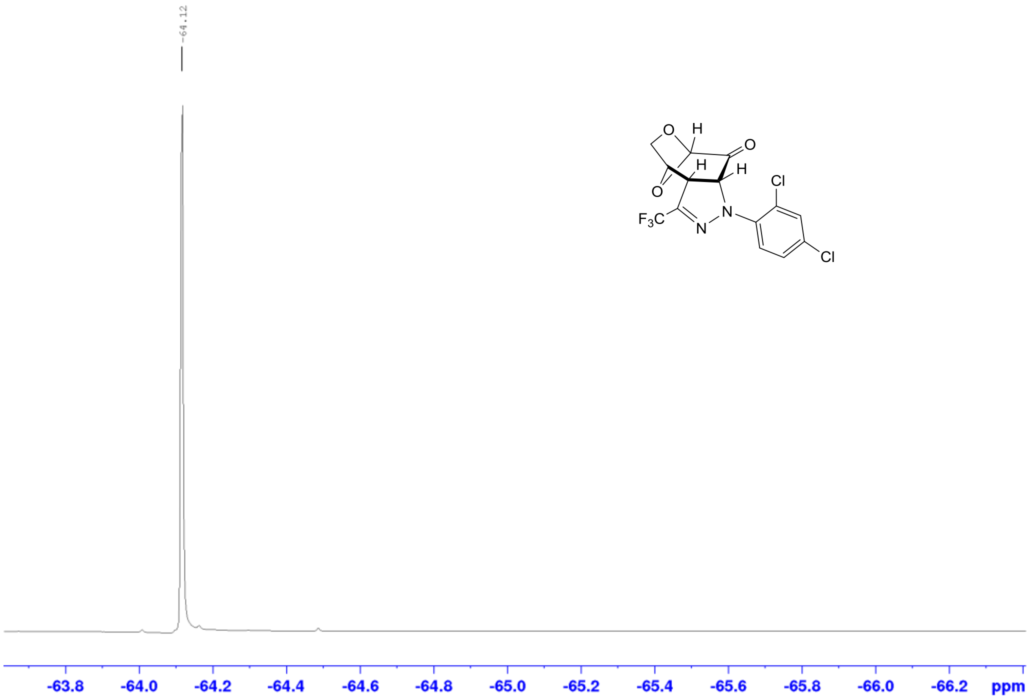

Fig. 8c. The ^{19}F NMR spectrum of (3+2)-cycloadduct *exo*-**11h**.

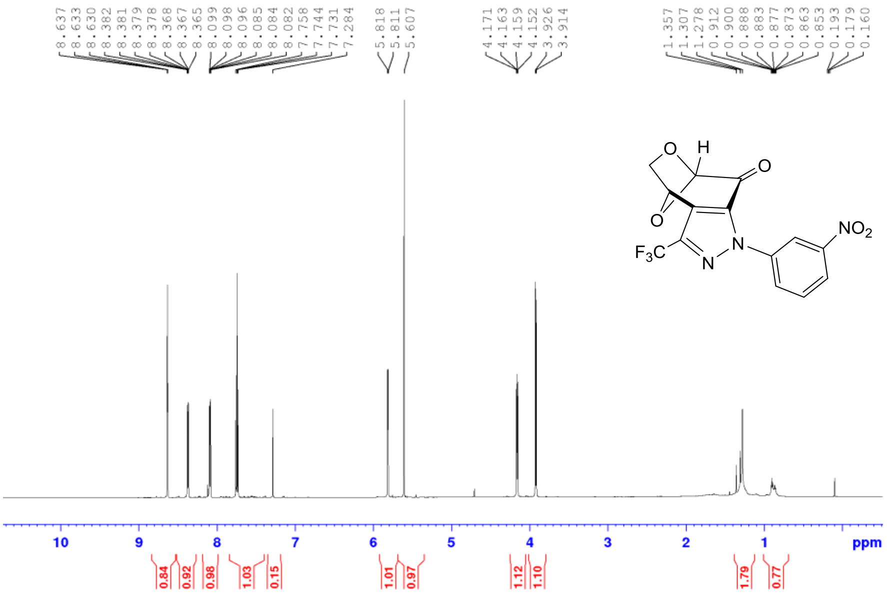

Fig. 9a. The ^{1}H NMR spectrum of fused pyrazole **12a**.

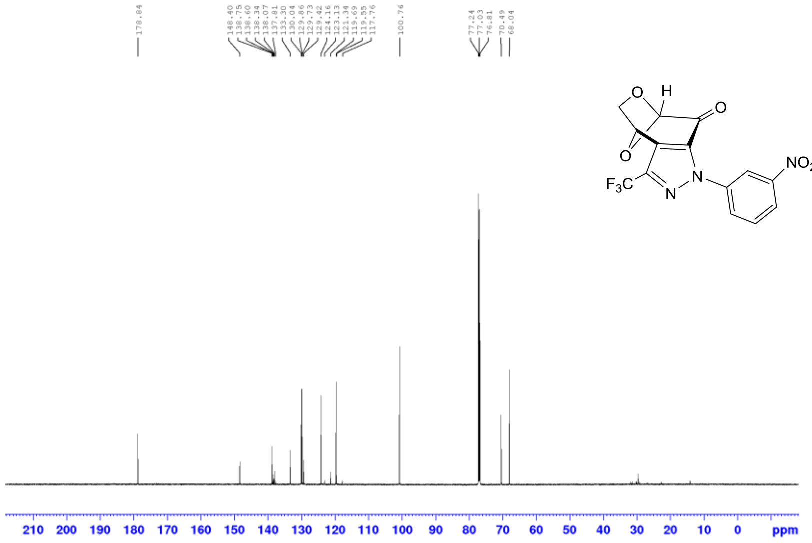

Fig. 9b. The ^{13}C NMR spectrum of fused pyrazole **12a**.

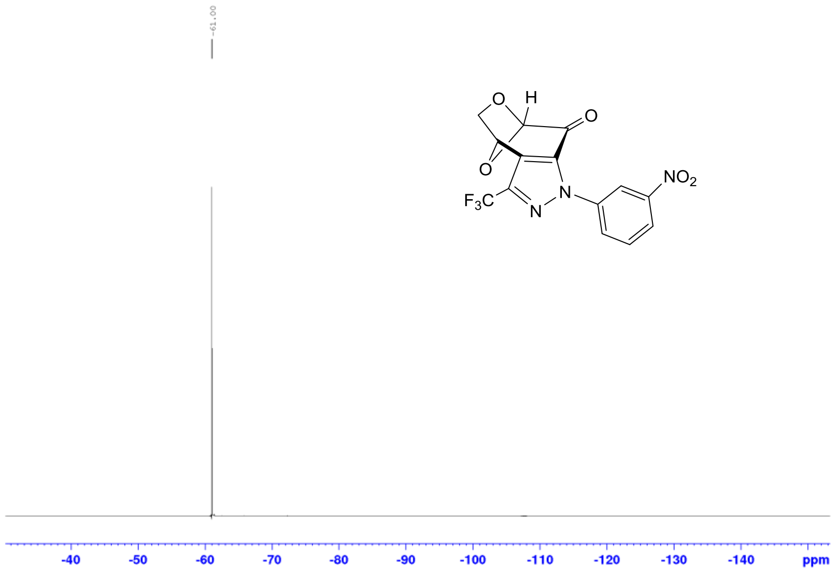

Fig. 9c. The ^{19}F NMR spectrum of fused pyrazole **12a**.

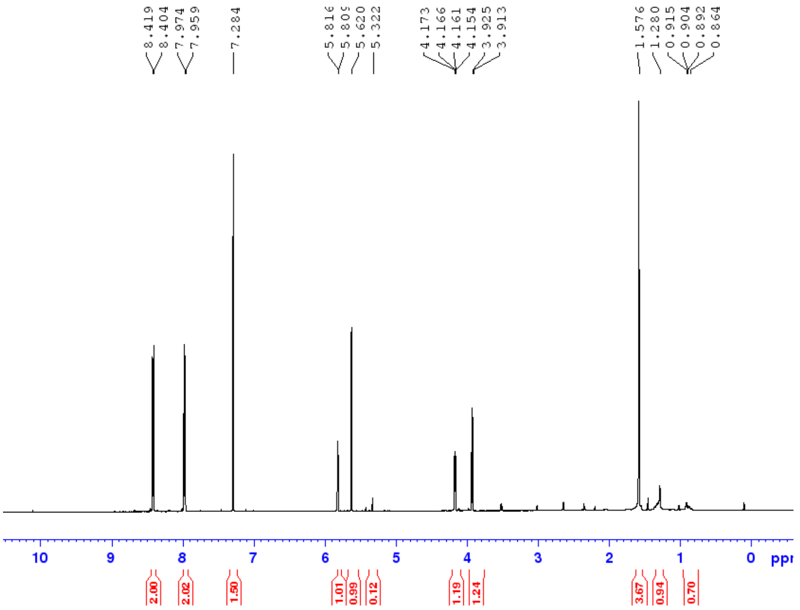

Fig. 10a. The ^{1}H NMR spectrum of fused pyrazole **12b**.

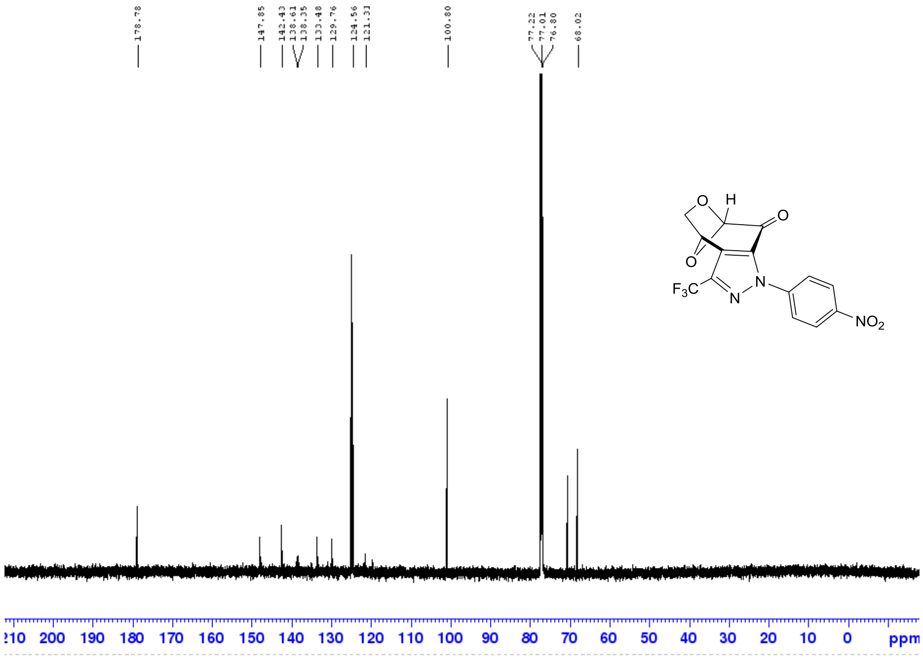

Fig. 10b. The ^{13}C NMR spectrum of fused pyrazole **12b**.

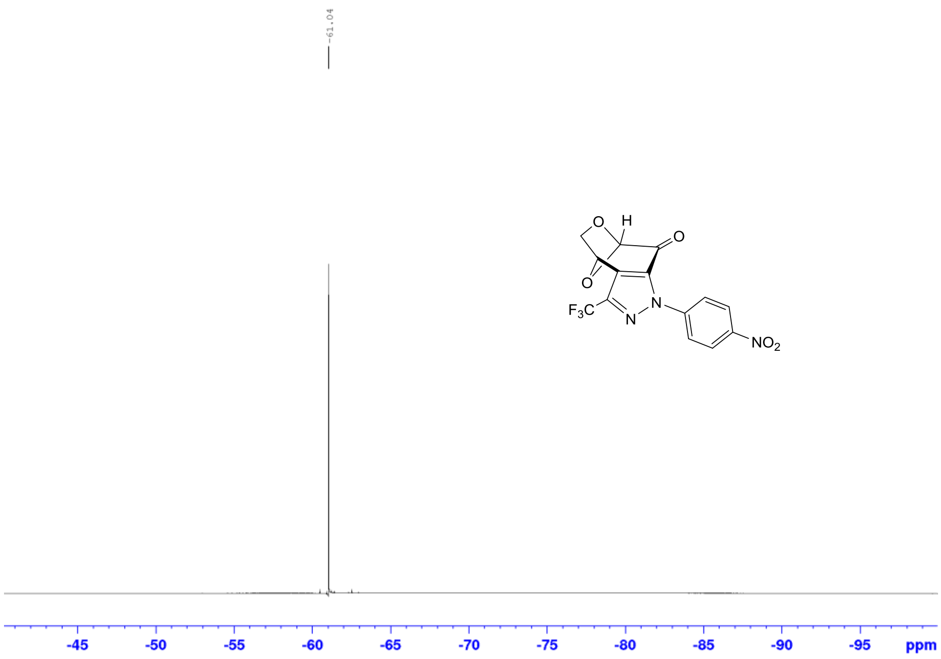

Fig. 10c. The ^{19}F NMR spectrum of fused pyrazole **12b**.

Section 4: *X-Ray structure determination of polycylic pyrazole **7** and (3+2)-cycloadduct (pyrazoline) exo-**11a***

Description of X-ray data collection experiments:

Polycylic pyrazole 7: X-ray diffraction data were collected at room temperature on an XtaLAB Synergy, Dualflex, HyPix diffractometer. Integration of the intensities and corrections for Lorentz effects, polarization effects, and analytical absorption were performed with CrysAlis PRO [1]. Using Olex2 [2], the structure was solved with the SHELXT [3] structure solution program using Intrinsic Phasing and refined with the SHELXL [4] refinement package using Least Squares minimization. The hydrogen atoms were introduced in the calculated positions with an idealized geometry and constrained using a rigid body model with isotropic displacement parameters equal to 1.2 of the equivalent displacement parameters of their parent atoms.

Polycylic pyrazoline *exo*-11a: X-ray diffraction data were collected on an XtaLAB Synergy, Dualflex, Pilatus 300K diffractometer. The crystal was kept at 99.97(13) K during data collection. Using Olex2 [2], the structure was solved with the SHELXT [3] structure solution program using Intrinsic Phasing and refined with the SHELXL [4] refinement package using Least Squares minimisation. The hydrogen atoms were introduced in the calculated positions with an idealized geometry and constrained using a rigid body model with isotropic displacement parameters equal to 1.2 of the equivalent displacement parameters of their parent atoms.

Table S1 Crystal data and structure refinement for pyrazole **7** and pyrazoline *exo*-**11a**.

Compound	pyrazole 7	pyrazoline *exo*-11a
Formula	$C_{19}H_{14}N_2O_3$	$C_{15}H_{13}F_3N_2O_3$
$D_{calc.}$/ g cm^{-3}	1.391	1.501
m/mm^{-1}	0.783	1.138
Formula Weight	318.32	652.55
Colour	light orange	colourless
Shape	prism	block
Size/mm^3	0.50.3 × 0.3	0.4 × 0.2 × 0.1
T/K	293(2)	100(2)
Crystal System	monoclinic	triclinic
Flack Parameter	-0.06(11)	-
Hooft Parameter	-0.02(7)	-
Space Group	$P2_1$	*P-1*
a/Å	5.58098(4)	8.52940(10)
b/Å	14.09945(13)	9.1083(2)
c/Å	9.65933(7)	10.5339(2)
α/°	90	103.055(2)
β/°	91.4502(6)	97.9850(10)
γ/°	90	111.055(2)
V/Å^3	759.837(10)	721.86(3)
Z	2	1
Z'	1	2
Wavelength/Å	1.54184	1.54184
Radiation type	CuK$_a$	CuK$_a$
Theta$_{min}$/°	4.579	4.441
Theta$_{max}$/°	76.576	78.767
Measured Refl.	20025	48427
Independent Refl.	2769	5558
Reflections with I > 2(I)	2716	5508
R_{int}	0.0431	0.0595
Parameters	218	418
Restraints	1	3
Largest Peak	0.141	0.169
Deepest Hole	-0.111	-0.152
GooF	1.056	1.035

wR_2 (all data)	0.0758	0.0597
wR_2	0.0752	0.0595
R_1 (all data)	0.0290	0.0237
R_1	0.0284	0.0234
CCDC	2278130	2083331

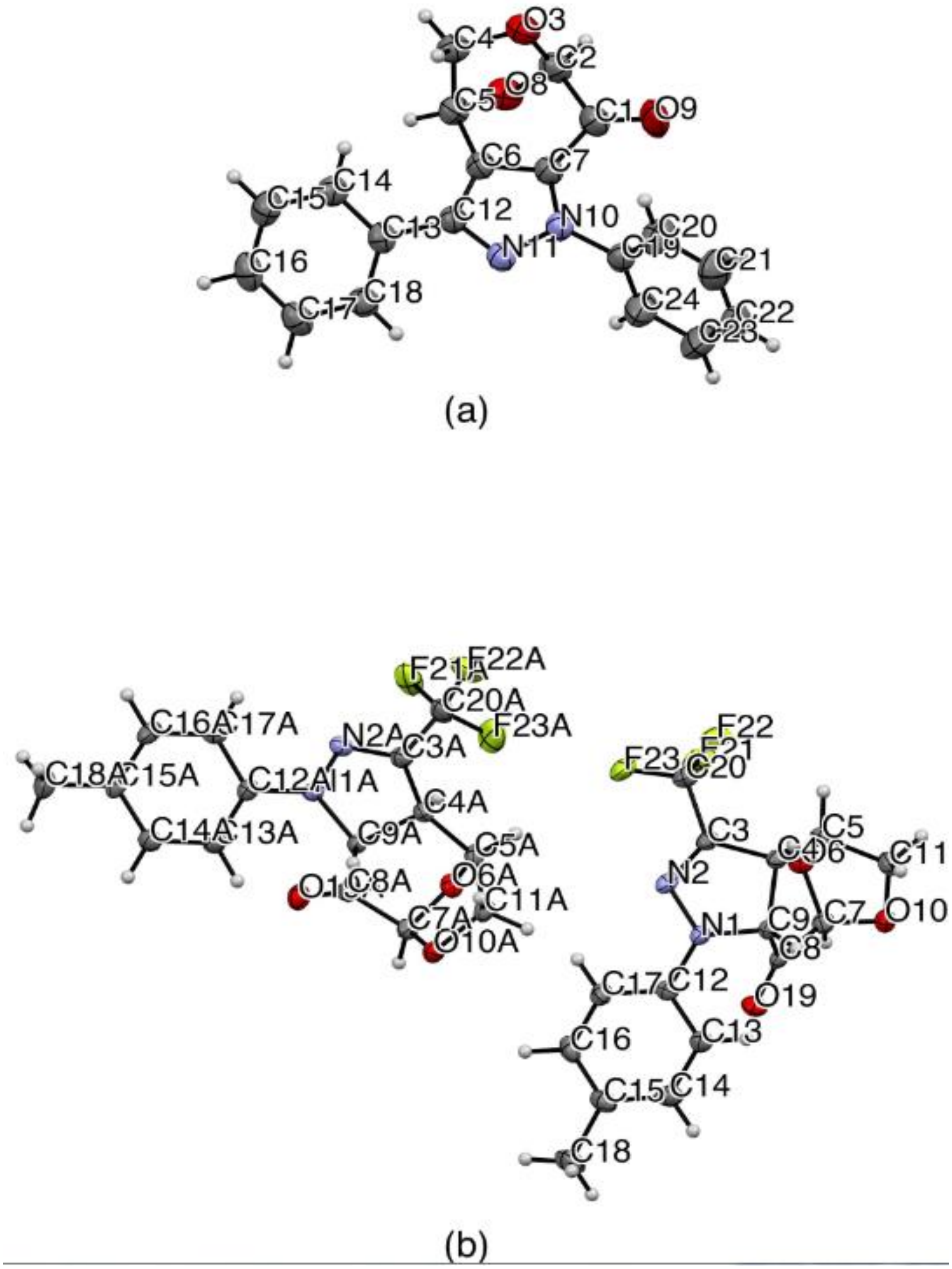

Fig. S1. ORTEP molecular graphs with crystallographic labelling scheme for polycyclic pyrazole **7**, (a), and polycylic pyrazoline *exo*-**11a**, (b). Ellipsoids drawn with 50% probability. In the case of (b) there are two molecules in the asymmetric cell unit.

Section 5: *DFT calculations*

All calculations were performed using the GAUSSIAN 16, B.01 package of programs [5]. The structures were fully optimized first using the B3LYP/6-31G(d)[6,7]+GD3BJ [8,9] method, then the PBE1PBE/def2tzvp[10–14]+GD3BJ [8,9] functional including the PCM-solvent sphere for dichloromethane [15]. Zero point vibrational energies and free enthalpy contributions were determined analytically.

Table S2: Total energies (E_{tot}) and Gibbs free energies (G_{298}) [a.u.], of cycloadducts **10**, respectively, the respective transition states **TS-10** and the relative energies with respect to the sum of the two educts diphenylnitrilimine **9** and LGO (**6**) (last column). Method: PBE1PBE/def2tzvp[6–10]+GD3BJ + PCM(CH_2Cl_2) [kcal/mol].

Species	E_{tot} [a.u.]	E_{rel} [kcal/mol]	G_{298} [a.u.]	E_{rel} [kcal/mol]
**Ph,Ph-Nitrilimine 9**	-610,34803		-610,19287	
**LGO 6**	-457,56199		-457,47692	
**Sum**	-1.067,91001	0.00	-1.067,66979	0.00
**TS-exo-10**	-1.067,91123	-0,76	-1.067,64853	13,34
**Exo-10**	-1.068,00579	-60,10	-1.067,73492	-40,87
**TS-exo-10´**	-1.067,90389	3,84	-1.067,64006	18,66
**Exo-10´**	-1.068,00544	-59,88	-1.067,73358	-40,03
**TS-Endo-10**	-1.067,91093	-0,57	-1.067,64793	13,72
**Endo-10**	-1.068,00833	-61,70	-1.067,73788	-42,73
**TS-Endo-10´**	-1.067,90307	4,36	-1.067,63912	19,24
**Endo-10´**	-1.068,00991	-62,69	-1.067,73812	-42,88

Gaussian Archive Entries

(Total energies (a.u.), number of imaginary frequencies (for transition states: imaginary frequencies), coordinates)

Diphenylnitrilimine 9

HF=-610.348027 a.u.(0)

1\1\GINC-R10N03\FOpt\RPBE1PBE\def2TZVP\C13H10N2\WURTHWE\08-Jun-2023\0\
\# pbe1pbe/def2tzvp Opt=(readfc,maxstep=2) geom=check guess=read Pop=NBO Freq emp=gd3bj scrf=(solvent=dichloromethane)\\ Educt Diphenylnitrilimine 9\\0,1\
C,0.1321101327,0.2699187443,-0.0033136896\C,0.0598612695,-0.2265099385,1.3029273165\
C,1.247436753,-0.4219600309,2.0169855359\C,2.468652662,-0.1288304006,1.4393012726\
C,2.5388939754,0.3646258196,0.1406241482\C,1.3631084048,0.5597730563,-0.5706930869\
N,-1.1235264845,-0.5479785099,1.9620741756\N,-2.1839172919,-0.3685586953,1.3114592921\
C,-3.220093333,-0.2424358438,0.8063350927\C,-4.4905477736,-0.097062291,0.2116057096\
C,-4.6028485432,0.388906886,-1.0972389552\C,-5.8539082193,0.5268462418,-1.6690457835\

C,-6.9964888325,0.1874459247,-0.9555510443\C,-6.8841330225,-0.294403081,0.3423629361\
C,-5.642028396,-0.4396512804,0.9321062052\H,1.1875166531,-0.8066596631,3.0285898828\
H,-0.7740368752,0.4296250858,-0.576229698\H,1.4009363269,0.9438554702,-1.5842876509\
H,3.4974198594,0.5933890797,-0.3093112731\H,3.377708721,-0.2871233824,2.0089435253\
H,-5.551581999,-0.8149933895,1.9436116065\H,-7.77260373,-0.5603223776,0.9024919511\
H,-7.9732282231,0.2983938741,-1.4106203066\H,-5.9368612259,0.9030702367,-2.6816632843\
H,-3.710932294,0.6527708799,-1.6513474143\\Version=ES64L-G16RevB.01\State=1-A\HF=-
610.348027\RMSD=1.449e-09\RMSF=2.048e-06\Dipole=-1.8941462,0.2752057,-1.0419338\
Quadrupole=8.6898549,-8.0289492,-0.6609057,1.1311401,-0.0430444,-3.2673342\PG=C01
[X(C13H10N2)]\\@

LGO 6
HF=-457.5619875 a.u. (0)
1\1\GINC-R02N38\FOpt\RPBE1PBE\def2TZVP\C6H6O3\WURTHWE\26-Feb-2022\0\\#
 pbe1pbe/def2tzvp Opt=readfc geom=check guess=read Pop=NBO Freq emp=gd
 3bj scrf=(solvent=dichloromethane)\\ Educt LGO\\0,1\
C,0.0327848381,0.023883915,4.5042260487\C,0.3420260204,0.6046260467,5.6670244356\
C,-0.6771073946,-1.2588730192,4.529972005\C,-0.9909336554,-1.7526509909,5.9434957913\
O,0.0896326786,-1.4979892766,6.7852418118\C,-0.0011439714,-0.0836526882,6.950395301\
O,-2.0691954323,-0.9644980002,6.4292404121\C,-1.5009053628,0.055173466,7.2428526935\
O,-1.0461799341,-1.8612173511,3.5450140032\H,-1.9141715411,1.0242285034,6.9622839209\
H,-1.705406119,-0.1521555157,8.2959739177\H,0.6384339498,0.2159363354,7.7774283448\
H,-1.2491863123,-2.811983052,5.9567395668\H,0.8064417757,1.5836751547,5.7132473175\
H,0.2605923226,0.4681279858,3.5424307836\\Version=ES64L-G16RevB.01\State=1-A\
HF=-457.5619875\ RMSD=6.415e-09\RMSF=3.989e-06\Dipole=0.7303336,1.6842253,1.4168192\
Quadrupole=-1.3694419,3.5815779,-2.212136,1.5931348,-1.8586196,-1.0921572\PG=C01
[X(C6H6O3)]\\ @

TS-exo-10
HF=-1067.9112255 a.u. (1, -259.2075 cm^{-1})
1\1\GINC-R02N38\FTS\RPBE1PBE\def2TZVP\C19H16N2O3\WURTHWE\26-Feb-2022\0
 \\# pbe1pbe/def2tzvp Opt=(ts,noeigentest,readfc) geom=check guess=read
 Pop=NBO Freq emp=gd3bj scrf=(solvent=dichloromethane)\\TS-exo-10\\0,1\
C,0.0141468429,-0.0877937806,-0.0070461024\C,0.064129778,-0.0139953704,1.3858178177\
C,1.2950032773,-0.0431315715,2.0403104311\C,2.4625647815,-0.1588469894,1.3068710812\
C,2.41725834,-0.2322548476,-0.0800286617\C,1.1900939762,-0.1930135766,-0.7302543068\
N,-1.0718424266,0.0690395086,2.2021110851\N,-2.1839547454,0.0947370049,1.6413670001\
C,-3.2714233313,-0.3984834996,1.5111045353\C,-4.6030336895,0.0024307277,1.1475705353\
C,-2.9831785689,-2.2817237654,2.6888699857\C,-3.9130146079,-1.8999079098,3.8085504913\
O,-3.3300788911,-0.829341262,4.5488874841\C,-2.2982969676,-1.4832946635,5.2228676577\
C,-1.1741919454,-1.8939212085,4.2682803528\C,-1.6390398894,-2.2889412566,2.9542504931\
O,-2.8646023544,-2.6883308813,5.7215982248\C,-3.9800467306,-2.9863345964,4.888174403\
O,-0.0246532532,-1.9716831238,4.6667404461\H,-3.8893073463,-3.9960109769,4.4848068559\
H,-4.9006980096,-2.8989306349,5.470477793\H,-4.8948443879,-1.5887499369,3.4576430703\
H,-1.9285813544,-0.8696971145,6.0448191704\H,-3.3843542392,-2.8678047214,1.8698994298\
H,-0.9152772569,-2.6889977446,2.2546755817\H,1.3114586473,0.0075438987,3.1218958441\
H,-0.9421373441,-0.0539566222,-0.5159961851\H,1.1480704457,-0.2434149146,-1.8123554625\
H,3.3337261236,-0.31451629,-0.6520612529\H,3.4160728634,-0.1884201683,1.8213127993\
C,-5.501995788,-0.9435917336,0.6538975752\C,-6.7904145957,-0.5644483939,0.3185031219\
C,-7.1919690734,0.75387256,0.4842811382\C,-6.3027980568,1.6978885065,0.984234633\
C,-5.0124445791,1.3292898505,1.3167905843\H,-5.1845418003,-1.9717121147,0.5298712003\
H,-7.4838335022,-1.2999434725,-0.0707663643\H,-8.2022829618,1.0477656796,0.2253616849\
H,-6.6182672933,2.7260624184,1.1151997098\H,-4.3127385742,2.0569106015,1.70937559
\\Version=ES64L-G16RevB.01\State=1-A\HF=-1067.9112255\RMSD=1.491e-09\RMSF=1.426e-

06\Dipole=-3.1391052,0.3108379,-2.5328347\Quadrupole=11.0607038,-1.2552256,-9.8054782, 3.1685341, -3.7015631,4.9016247\PG=C01 [X(C19H16N2O3)]\\@

Exo-10

HF=-1068.005787 a.u. (0)
1\1\GINC-R03N28\FOpt\RPBE1PBE\def2TZVP\C19H16N2O3\WURTHWE\26-Feb-2022\
0\\# pbe1pbe/def2tzvp Opt=readfc geom=check guess=read Pop=NBO Freq emp=gd3bj
scrf=(solvent=dichloromethane)\\exo-10\\0,1\
C,0.0222834248,0.1168430717,0.0472756672\C,0.0175632463,0.0205460344,1.4286206424\
C,1.2234575624,0.0143330862,2.1359019441\C,2.426372084,0.10169926,1.4260868581\
C,2.4104288027,0.1884264721,0.045344033\C,1.2135867356,0.2002343515,-0.6594865591\
N,1.2297945725,-0.053372994,3.5143950888\C,0.0787838957,-0.0781409268,4.3929612161\
C,0.6790961155,0.412923777,5.7267308795\C,2.1463810883,0.1481725561,5.4615084737\
N,2.3846833963,-0.0504750956,4.214154095\C,-0.5075704752,-1.4924463361,4.4956519685\
C,-1.0545689954,-1.8348805309,5.8714317222\O,0.0231635741,-1.678214643,6.7533588921\
C,0.063291697,-0.2655970671,6.9421622734\O,-2.043063767,-0.9089300899,6.2494654506\
C,-1.4152992365,0.0556649011,7.0962422548\O,-0.4873626207,-2.2786115952,3.5879721412\
C,3.2191595169,0.211216385,6.442793242\H,-1.6787016297,1.060184272,6.7629099227\
H,-1.7503567224,-0.0907315216,8.125323238\H,0.6401839223,-0.0694531827,7.8434028075\
H,-1.4312701373,-2.8581577128,5.9095676684\H,0.5352700639,1.4954078884,5.8327995691\
H,-0.7160269968,0.5826793747,4.0375439236\H,-0.925698219,-0.0726497877,1.9502196356\
H,3.3611679011,0.1031468492,1.969077767\H,3.3533080059,0.2524184932,-0.4865179427\
H,1.2098901767,0.270503103,-1.7402991989\H,-0.9245974813,0.1179266244,-0.4808780151\
C,4.415228733,-0.4799169949,6.2268192089\C,5.4479203016,-0.3988278079,7.1427932081\
C,5.3066278823,0.3681691028,8.2937446461\C,4.123937915,1.0570350029,8.5180789785\
C,3.0860772558,0.9781096362,7.602503502\H,4.5161625752,-1.0867379227,5.3352878692\
H,6.3676991794,-0.9436954947,6.9639667916\H,6.1150019377,0.4251380664,9.0131709281\
H,4.0076325059,1.6630049847,9.4090455028\H,2.1794744168,1.5445614103,7.7806815029
\\Version=ES64L-G16RevB.01\State=1-A\HF=-1068.005787\RMSD=3.448e-09\RMSF=1.151e-06\Dipole=-0.3760502,1.625793,2.1034114\Quadrupole=3.3140055,-7.1950659,3.8810604,-6.1334024,0.0308759,3.13264\PG=C01 [X(C19H16N2O3)]\\@

TS-exo-10´

HF=-1067.9038944 a.u (1, -279.3792cm^{-1})
1\1\GINC-R09N18\FTS\RPBE1PBE\def2TZVP\C19H16N2O3\WURTHWE\01-Mar-2022\0
\\# pbe1pbe/def2tzvp opt=(ts,noeigentest,readfc) geom=check guess=read Pop=NBO Freq emp=gd3bj
scrf=(solvent=dichloromethane)\\TS-exo-10´\\0,1\
C,-0.1073586336,0.2556779154,0.5144584707\C,-0.1795579012,-0.3164817253,1.7845673025\
C,0.9282370356,-0.9974273852,2.3028013977\C,2.0872855533,-1.0946239826,1.5563465018\
C,2.1535155317,-0.5225121032,0.2910959138\C,1.0569399947,0.1513597217,-0.2274096299\
C,-1.3936963809,-0.2171498817,2.5494935419\C,-3.3984122086,-0.001014433,1.542561551\ ´
C,-4.2673493856,0.0017625929,2.6025430777\N,-2.7169198743,-0.7150194204,4.4286213463\
N,-1.7647894064,-0.4437979593,3.6710486019\C,-3.4650602758,-1.171146138,0.5836606362\
O,-4.8370557133,-1.574387381,0.4493277154\C,-5.0628837542,-2.1869389921,1.684043392\
C,-5.179906745,-1.1156342175,2.7765013917\C,-2.88585693,-2.4870406265,1.1049252771\
O,-3.9214430694,-2.9861726393,1.9432900468\C,-2.9415154444,0.1359336877,5.5191250644\
C,-4.0165525364,-0.1878448767,6.3457465897\C,-4.3227024946,0.6207602371,7.4258829091\
C,-3.5566505576,1.7472883653,7.7006242632\C,-2.4801382424,2.0622029066,6.8810067667\
C,-2.1696503904,1.2664061235,5.7911505392\O,-6.0178691528,-1.2107772668,3.6537889321\
H,-1.9689538019,-2.3900093209,1.6812270713\H,-2.7213244092,-3.1736481725,0.2699144339\
H,-3.0909215118,-0.9071364842,-0.4033309772\H,-5.9633132982,-2.8018491503,1.6439714965\
H,-3.0059055178,0.9364429278,1.1702099578\H,-4.4378294787,0.8867745052,3.2029685534\
H,-4.6079437119,-1.0641530666,6.1113861701\H,-1.326947907,1.5111686548,5.1550022386\
H,-1.8745358069,2.9360140221,7.093027211\H,-3.7941349219,2.3741187134,8.5518442554\
H,-5.1641026385,0.3683545974,8.0607531699\H,0.8656325099,-1.4436316107,3.2878322215\

H,2.9434389771,-1.6214441317,1.9604394355\H,3.0634139124,-0.6041618049,-0.2915741282\
H,1.1082920264,0.5979181034,-1.212891849\H,-0.9622246698,0.7846221902,0.1126057205
\\Version=ES64L-G16RevB.01\State=1-A\HF=-1067.9038944\RMSD=3.130e-09\RMSF=1.537e-06\Dipole=3.5225267,1.1725002,-1.5884041\Quadrupole=-3.2045383,-3.8502462,7.0547845,-4.2721262,-7.5872783,2.2075131\PG=C01 [X(C19H16N2O3)]\\@

Exo-10´
HF=-1068.0054398 a.u. (0)
1\1\GINC-R09N14\FOpt\RPBE1PBE\def2TZVP\C19H16N2O3\WURTHWE\01-Mar-2022\
0\\# pbe1pbe/def2tzvp opt=readfc geom=check guess=read Pop=NBO Freq em
p=gd3bj scrf=(solvent=dichloromethane)\\exo-10´\\0,1\
C,-0.0147942909,-0.0196225783,0.070421399\C,0.003965685,0.0063156052,1.4581457923\
C,1.2218977807,-0.0005709842,2.1357569726\C,2.4125801929,-0.0423712362,1.4042674355\
C,2.3748887175,-0.0749526163,0.0229094731\C,1.162770685,-0.0608289963,-0.6579176996\
N,1.2761904215,0.0443514304,3.5296767175\C,0.105822856,0.0412174568,4.4097923663\
C,0.7722682706,-0.1170725636,5.7885983734\C,2.0945260011,-0.716167871,5.3898889709\
N,2.3327196931,-0.5533300042,4.1391616073\C,-0.7618596517,1.2971210559,4.3247420331\
C,-0.359283075,2.4459871665,5.2421210626\O,-0.2887686222,1.911518979,6.5364500596\
C,0.9630940852,1.2290152588,6.5068555991\O,0.9160288531,2.9288245662,4.9275389394\
C,1.8395964422,2.235203565,5.7696552548\O,-1.7648089601,1.3466258157,3.6615100191\
C,3.0587776798,-1.31186008,6.3021356925\H,2.619961181,1.7757594383,5.1635944512\
H,2.2875074486,2.9464401309,6.4660970275\H,1.2869249912,1.0683692354,7.5337732946\
H,-1.1027 642456,3.2430511913,5.2006595522\H,0.1744787419,-0.7599141505,6.434297144\
H,-0.5381804495,-0.8177040093,4.1788802073\H,-0.9294265572,0.0370156938,2.0023796632\
H,3.3569041866,-0.0518061854,1.9310842125\H,3.3073962605,-0.1071856828,-0.5293046133\
H,1.1401150989,-0.0814346617,-1.7407548986\H,-0.9709065553,-0.0133811544,-0.4406459898\
C,4.3469498043,-1.6437873416,5.8674725722\C,5.2552335988,-2.2120029606,6.7394544464\
C,4.9006629388,-2.4601154798,8.0619719966\C,3.6273024718,-2.1351469432,8.5026129053\
C,2.7121948545,-1.5650370594,7.6303874608\H,4.6218409377,-1.4464808924,4.8387084189\
H,6.2500151995,-2.4626309433,6.3895414598\H,5.6164908131,-2.904454356,8.7434355104\
H,3.3410425039,-2.3263601884,9.5301563646\H,1.7175033235,-1.3269485818,7.9885938378
\\Version=ES64L-G16RevB.01\State=1-A\HF=-1068.0054398\RMSD=2.392e-09\RMSF=2.229e-06\Dipole=0.9524871,-0.7086787,1.7436886\Quadrupole=-1.0491697,-6.5695428,7.6187125,
1.5043198,0.3638438,-1.6152789\PG=C01 [X(C19H16N2O3)]\\@

TS-endo-10
HF=-1067.9109277 a.u (1, -269.7865cm^{-1})
1\1\GINC-R02N39\FTS\RPBE1PBE\def2TZVP\C19H16N2O3\WURTHWE\26-Feb-2022\0\\#
pbe1pbe/def2tzvp Opt=(ts,noeigentest,readfc) geom=check guess=read Pop=NBO Freq emp=gd3bj
scrf=(solvent=dichloromethane)\\TS-endo-10\\0,1\
C,-0.0553627327,0.0062639971,0.0841032819\C,-0.0211355105,0.0203824284,1.4791064117\
C,1.1978549595,0.1990432113,2.1339472586\C,2.3604742094,0.3716563893,1.4033595522\
C,2.3281454828,0.3557150307,0.0143339631\C,1.1164570358,0.1678976681,-0.6370964757\
N,-1.1596439595,-0.1117968573,2.2865881185\N,-2.2568340817,-0.3154266445,1.6922651152\
C,-3.395703405,0.0280842878,1.6062490246\C,-4.7327356051,-0.3890336654,1.3358879937\
C,-2.9370282612,2.0637482775,2.848044268\C,-4.1142485228,1.9023663074,3.6817420026\
C,-3.8359231578,1.2318920356,5.0288957936\O,-2.8161272374,0.2881059903,4.9134882625\
C,-1.6724127584,1.1229106718,4.7384503932\C,-1.7091964344,1.7309153114,3.3695488543\
C,-1.9213256561,2.1732869853,5.8278533911\O,-3.3425009688,2.2382493176,5.9015102669\
O,-5.2270753766,2.3242955291,3.4199735632\H,-1.5132126598,3.1542067374,5.5790638789\
H,-1.5236638136,1.8384578798,6.7891603011\H,-0.7776897148,0.5287151436,4.9053556015\
H,-4.7352823425,0.7772137458,5.4458612518\H,-0.8001966484,2.1852263982,2.9941118492\
H,-3.0309000581,2.667074135,1.9533718585\H,-0.9986818362,-0.1392095144,-0.4290809432\
H,1.0805741792,0.1489556866,-1.7205251672\H,3.2408040856,0.4830024077,-0.555311956\
H,3.3012855839,0.5120309367,1.9231308727\H,1.2204388647,0.1964670059,3.2175714817\

C,-5.6557705116,0.5227902624,0.821211146\C,-6.9537121853,0.1159549442,0.5689633406\
C,-7.3384408262,-1.190000305,0.8407877169\C,-6.4253056139,-2.0969735727,1.366409117\
C,-5.1245633478,-1.7034366313,1.6172674473\H,-5.3480797056,1.5432743226,0.635303764\
H,-7.6701039086,0.8214590343,0.1663261424\H,-8.3575524839,-1.5034013537,0.6469221965\
H,-6.7304199827,-3.1141459266,1.5806488106\H,-4.4027540237,-2.3992274719,2.0268617294
\\Version=ES64L-G16RevB.01\State=1-A\HF=-1067.9109277\RMSD=2.403e-09\RMSF=2.765e-06\Dipole=0.8585431,-0.8917173,-1.0970923\Quadrupole=7.9553893,-6.0037857,-1.9516035,14.5526467,11.2845756,-2.2612249\PG=C01 [X(C19H16N2O3)]\\@

Endo-10
1\1\GINC-R03N30\FOpt\RPBE1PBE\def2TZVP\C19H16N2O3\WURTHWE\26-Feb-2022\
 0\\# pbe1pbe/def2tzvp Opt=readfc geom=check guess=read Pop=NBO Freq emp=gd3bj
scrf=(solvent=dichloromethane)\\Endo-10\\0,1\
C,-0.0385866195,0.0134478939,0.0336329811\C,-0.0428775431,-0.0028714845,1.4324599226\
C,1.1760495406,-0.1207370428,2.1115979171\C,2.3582518269,-0.2131083091,1.4017008028\
C,2.3654755485,-0.1903440521,0.0115171488\C,1.158476934,-0.0785530188,-0.6601682462\
N,-1.2282309 112,0.0976099232,2.1340764664\C,-2.5497059118,0.2506890677,1.5337938406\
C,-3.486849731,-0.0515514905,2.7261461013\C,-2.4857641072,-0.2532168725,3.8577333268\
N,-1.273550247,-0.1736055998,3.4486817263\C,-2.717923856,1.6677350025,0.9879404337\
O,-2.9150538894,2.5630945856,2.078395986\C,-4.2673803245,2.3865847793,2.3593310018\
C,-4.4993680558,1.0408228304,3.0351471298\C,-3.9896889435,1.8639254994,0.1668327182\
O,-4.9386099571,2.3420618025,1.1197697908\C,-2.80187321,-0.5655739605,5.2449900328\
O,-5.4587906172,0.8575818155,3.7375741844\H,-4.3465910503,0.9385008057,-0.2902547166\
H,-3.8558171158,2.6225854852,-0.6056229691\H,-1.8199083145,1.979076977,0.4581913001\
H,-4.6475701969,3.2065139532,2.9692359154\H,-2.6904435897,-0.4754523785,0.7278631633\
H,-4.0591090388,-0.9700067387,2.5739780906\H,1.1768657587,-0.1413047143,3.1924951599\
H,3.2913971083,-0.303435542,1.946555072\H,3.2970011778,-0.260592584,-0.5364411492\
H,1.1376568502,-0.0656206186,-1.7440304785\H,-0.963005808,0.085931664,-0.5242844056\
C,-1.8457827082,-0.345366462,6.24294235\C,-2.1119842185,-0.6798357097,7.557293236\
C,-3.3377757368,-1.2370813278,7.9045010918\C,-4.2932876528,-1.4542272838,6.9233800392\
C,-4.0318082826,-1.1194431643,5.6035902288\H,-0.8950484562,0.0961228957,5.9705740778\
H,-1.3623756991,-0.4992667865,8.3191759455\H,-3.5468652544,-1.4944549569,8.9362831193\ H,-5.2521913743,-1.8866867497,7.1845607617\H,-4.7912713635,-1.2842036046,4.8513165232
\\Version=ES64L-G16RevB.01\State=1-A\HF=-1068.0083326\RMSD=5.509e-09\RMSF=8.401e-07\Dipole=0.2010976,-0.4068218,-1.3058426\Quadrupole=-8.2800911,-5.5565951,13.8366863,4.9710995,2.7912199,-1.6 901786\PG=C01 [X(C19H16N2O3)]\\@

TS-endo-10´
HF=-1067.9030681 a.u. (1, -299.6172cm^{-1})
1\1\GINC-R10N19\FTS\RPBE1PBE\def2TZVP\C19H16N2O3\WURTHWE\01-Mar-2022\0
 \\# pbe1pbe/def2tzvp opt=(ts,noeigentest,readfc) geom=check guess=read Pop=NBO Freq emp=gd3bj
scrf=(solvent=dichloromethane)\\TS-endo-10´\\0,1\
C,0.1269010574,0.5351805182,0.2449258952\C,0.0016811981,-0.1434317758,1.4570410542\
C,1.0611892918,-0.9310365432,1.929134797\C,2.2281213324,-1.0242863489,1.1956597542\
C,2.3479016356,-0.3535900115,-0.0163795602\C,1.2962504975,0.4179111686,-0.4888190545\
C,-1.1972165174,-0.0470869753,2.240116933\C,-3.245917387,0.6014493712,1.3216610371\
C,-4.1161082244,0.3211165354,2.3517349217\N,-2.7676049326,-0.8793830405,3.7562826801\
N,-1.6773840096,-0.5529559027,3.218001329\C,-3.0551263227,2.0081108934,0.9571987184\
C,-3.7546802587,3.0093285845,1.8844704482\O,-5.0092943315,2.5282890756,2.2549678937\
C,-4.6449286446,1.4736411713,3.1644228539\C,-3.6054806362,2.2187901949,4.005872394\
O,-2.9972190435,3.1080103991,3.0732345398\C,-2.8736530651,-0.7705234149,5.1468911469\
C,-4.1135733624,-1.0736082261,5.7098895097\C,-4.2987555618,-0.968414648,7.0768667779\
C,-3.2517880773,-0.5749286773,7.9018799049\C,-2.0142155824,-0.2867353509,7.3424774139\
C,-1.819402842,-0.3788386953,5.9741139874\O,-2.4591578337,2.4004471288,-0.0265137501\
H,-3.8460321308,3.982405985,1.4003581017\H,-4.1056938089,2.7968279633,4.7868474883\

H,-5.5285391317,1.1970886583,3.7343210142\H,-2.8447968462,1.5856211022,4.4523299681\
H,-4.6628207623,-0.6086932532,2.3710057205\H,-3.0649397063,-0.1260071111,0.5370186097\
H,-0.8523082209,-0.1532768534,5.5397522967\H,-1.1889804405,0.0152796316,7.9773344588\
H,-3.398518639,-0.4985323859,8.9724318747\H,-5.2680543485,-1.2002096344,7.5029815772\
H,-4.924546103,-1.3854135585,5.0619655064\H,0.9569174245,-1.4570137904,2.8701897745\
H,3.0468359929,-1.6295618818,1.5665228383\H,3.2630575708,-0.4346847847,-0.591025959\
H,1.3874483505,0.9400305529,-1.4338256015\H,-0.6938488143,1.1427439249,-0.1150193226
\\Version=ES64L-G16RevB.01\State=1-A\HF=-1067.9030681\RMSD=2.920e-09\RMSF=4.909e-07\Dipole=0.5134212,-1.4206796,1.1807791\Quadrupole=10.960317,-11.4886739,0.5283568,-0.9900833,-8.286595,5.231004\PG=C01 [X(C19H16N2O3)]\\@

Endo-10´
HF=-1068.0099137 a.u. (0)
1\1\GINC-R07N08\FOpt\RPBE1PBE\def2TZVP\C19H16N2O3\WURTHWE\01-Mar-2022\
0\\# pbe1pbe/def2tzvp opt=readfc geom=check guess=read Pop=NBO Freq emp=gd3bj scrf=(solvent=dichloromethane)\\endo-10´\\0,1\
C,0.0014328432,-0.1155878371,0.0287742469\C,-0.0174523316,-0.0163349487,1.4233788108\
C,1.1803299431,0.2286477664,2.1036191122\C,2.3609446657,0.3642046088,1.3959443863\
C,2.3837660512,0.2615561129,0.0103617222\C,1.194514458,0.0226363172,-0.6619237473\
N,-1.2083145122,-0.1391015473,2.1141285723\C,-2.4674203532,-0.6143151979,1.5668793715\
C,-3.4389443534,-0.319204194,2.7258877776\C,-2.453776495,-0.1850875558,3.8817257548\
N,-1.247724709,-0.0520518589,3.4588517693\C,-4.5234672691,-1.3661578782,2.890155682\
C,-4.1710727899,-2.7490787651,2.3610907063\O,-3.7377658547,-2.5847079528,1.0379318969\
C,-2.4066607127,-2.1107256474,1.2140136922\C,-1.9077794748,-3.0050429214,2.3373279782\
O,-3.0930148061,-3.2782284827,3.0883185342\O,-5.6058430273,-1.1199923292,3.3562545698\
C,-2.7770636961,-0.1056587489,5.29876903\H,-5.0357656541,-3.4128135123,2.3971072457\
H,-1.5100119298,-3.941888522,1.9421807654\H,-1.8640906532,-2.2653728867,0.2834569327\
H,-1.163683156,-2.5341644445,2.9790579662\H,-2.7481844381,-0.0582394516,0.6721529808\
H,-3.9483811872,0.6405410819,2.5876008801\H,1.168061273,0.3105114961,3.1814937141\
H,3.2796810149,0.552467916,1 .9401894515\H,3.3133404654,0.3673806313,-0.535160058\
H,1.1866808291,-0.0572370059,-1.7430290511\H,-0.9124953908,-0.2896181169,-0.5247098193\
C,-1.7862894804,-0.3545602127,6.2550058709\C,-2.0704841211,-0.2483853746,7.6032335101\
C,-3.3483791833,0.1030763017,8.0247555949\C,-4.33751469,0.3468126572,7.084263953\
C,-4.0584417699,0.241819667,5.7299518396\H,-0.7952700985,-0.6387367912,5.9231887997\
H,-1.2941784993,-0.4482328937,8.3328336524\H,-3.5707051101,0.1805544571,9.0826210903\
H,-5.3360548041,0.62135107,7.4039750556\H,-4.8401448359,0.4330480422,5.0065470011
\\Version=ES64L-G16RevB.01\State=1-A\HF=-1068.0099137\RMSD=5.689e-09\RMSF=1.492e-06\Dipole=0.7017031,-0.003464,-0.7818829\Quadrupole=-5.0543871,-5.035222,10.0896091,-4.4348096,-0.0201988,1.7459613\PG=C01 [X(C19H16N2O3)]\\@

Table S3: Total energies (E_{tot}) and Gibbs free energies (G_{298}) [a.u.], of cycloadducts **11b**, respectively, the respective transition states **TS-11b** and the relative energies with respect to the sum of the two educts nitrilimine **1b** and LGO (**6**) (last column). Method: PBE1PBE/def2tzvp[6–10]+GD3BJ + PCM(CH_2Cl_2) [kcal/mol].

Species	E_{tot} [a.u.]	E_{rel} [kcal/mol]	G_{298} [a.u.]	E_{rel} [kcal/mol]
**CF_3-, Ph-Nitrilimine 1b**	-716,33812		-716,25747	
**LGO 6**	-457,56199		-457,47692	

**Sum**	-1.173,90010	0.00	-1.173,73439	0.00
**TS-11b**	-1.173,90030	-0,12	-1.173,71319	13,30
**11b**	-1.174,00553	-66,15	-1.173,81046	-47,73
**TS-11´b**	-1.173,89754	1,61	-1.173,70961	15,55
**11´b**	-1.174,00729	-67,26	-1.173,81183	-48,60
**TS-11b-a**	-1.173,89263	4,69	-1.173,70507	18,40
**11b-a**	-1.173,70507	-64,70	-1.173,80711	-45,64
**TS-11´b-a**	-1.173,89246	4,80	-1.173,70455	18,73
**11´b-a**	-1.174,00821	-67,84	-1.173,81205	-48,74

Gaussian Archive Entries

(Total energies (a.u.), number of imaginary frequencies (for transition states: imaginary frequencies), coordinates)

CF3-Nitrilimine **1b**
HF=-716.3381159 a.u. (0)
1\1\GINC-R09N42\FOpt\RPBE1PBE\def2TZVP\C8H5F3N2\WURTHWE\08-Jun-2023\0\
\# pbe1pbe/def2tzvp Opt=(readfc,maxstep=5) geom=check guess=read Pop=N
BO Freq emp=gd3bj scrf=(solvent=dichloromethane)\\Educt CF3,Ph-Nitrilimine \\0,1\
C,0.0874486531,0.1758029427,-0.0674226857\ C,0.0196793452,0.0952902607,1.3180373916\
C,1.1833673307,-0.0598760165, 2.0597689849\ C,2.4099494731,-0.1367028327,1.4229350597\
C,2.4706123532,-0.0573634234,0.0352075799\ C,1.3081951787,0.1029515091,-0.7157669311\
N,3.7579612964,-0.1550255816,-0.533068516\ N,3.8577526581,-0.1194778009,-1.7424941509\
C,4.0386694794,-0.2517603971,-2.9167264726\ C,4.299033139,0.8211140521,-3.9145005642\
F,3.2907541497,0.883715685,-4.7861974724\ F,4.4373390173,2.0403621288,-3.3764015061\
F,5.4175059641,0.5602846579,-4.5915027673\ H,3.3288979774,-0.2608519287,1.9828070614\
H,1.3570411106,0.1732247852,-1.7960344949\ H,-0.8184089187,0.300233573,-0.6488989612 \H,-
0.9394202361,0.1565396464,1.8180673866\ H,1.1358581095,-0.1223529702,3.1403769145
\\Version=ES64L-G16RevB.01\ State=1-A\ HF=-716.3381159\RMSD=5.604e-09\RMSF=7.652e-
07\Dipole=-0.9933324,0.003622,0.9377225\ Quadrupole=2.7803109,-4.533666,1.753355,-
0.1377972,-0.9958064,-0.4841338\PG=C01 [X(C8H5F3N2)]\\@

TS-11b
HF=-1173.9002998 a.u. (1, -246.0182 cm^{-1})
1\1\GINC-R01N22\FTS\RPBE1PBE\def2TZVP\C14H11F3N2O3\WURTHWE\26-Feb-2022
\0\\# pbe1pbe/def2tzvp Opt=(ts,noeigentest,readfc) geom=check guess=re
ad Pop=NBO Freq emp=gd3bj scrf=(solvent=dichloromethane)\\TS-Cycloaddition TS-11b\\0,1\
C,-0.0022489648,-0.1362501409,-0.015363428\C,-0.0758855897,0.0215364233,1.5081714194\
O,1.3095159997,0.0414483627,1.8402990367\C,1.8201189164,0.9087005047,0.872912184\
O,1.2113676822,0.5272960824,-0.3522850986\C,-0.6920582739,1.339634916,1.8938400797\
C,0.0496812321,2.4714724667,1.6871575996\C,1.3899365073,2.3526055878,1.1400495262\
O,2.0979921395,3.2854813322,0.807662904\N,1.1971617221,2.6424626597,3.9795409919\
N,0.3652846712,1.8383691888,4.3900029704\C,-0.5271085673,1.0705860025,4.1243665677\
C,-0.986437872,-0.1183631678,4.897768162\F,-2.0726239258,-0.6461431791,4.3298113115\
C,1.1764292729,3.9636583726,4.44443489\C,2.1635375791,4.8052797834,3.937219443\
C,2.1847377575,6.135581125,4.3170891834\C,1.2373971439,6.6236750533,5.2078646569\
C,0.2608385002,5.7757966186,5.71791876\C,0.2213704068,4.4461521823,5.3391579332\

F,-0.0524896999,-1.0729429631,4.9339538324\F,-1.2994941005,0.2035738335,6.1531443119\
H,-0.8415944476,0.326941462,-0.5359799374\H,0.0715950348,-1.1909004497,-0.2908335594\
H,-0.5445148857,-0.8297695627,1.9972553173\H,2.9039914665,0.8125639278,0.8064611759\
H,-1.7706020936,1.3906959199,1.9860071318\H,-0.3677223715,3.4638978131,1.8079094439\
H,2.8856856892,4.4039147069,3.2376165733\H,-0.5352676791,3.7813525875,5.7390229853\
H,-0.4743869927,6.1541553474,6.4182386102\H,1.2618176285,7.6638081741,5.5099870377\
H,2.9468653025,6.7943974645,3.9185667795\\Version=ES64L-G16RevB.01\State=1-A\ HF=-1173.9002998\RMSD=1.390e-09\RMSF=1.240e-06\Dipole=-1.6917675,0.1342766,1.1128651\
Quadrupole=0.0546538,7.2876483,-7.3423021,1.2459881,6.766373,11.9477327\
PG=C01 [X(C14H11F3N2O3)]\\@

11b
HF=-1174.0055261 a.u. (0)
1\1\GINC-R03N33\FOpt\RPBE1PBE\def2TZVP\C14H11F3N2O3\WURTHWE\26-Feb-2022\0\\ # pbe1pbe/def2tzvp Opt=readfc geom=check guess=read Pop=NBO Freq
emp=gd3bj scrf=(solvent=dichloromethane)\\Product 11b\\0,1\
O,-0.0503391585,3.8947402967,0.1295538564\C,-1.4140237161,3.6498679162,0.4794791832\
C,-1.8201137379,2.5511161888,-0.4904263976\O,-1.0428421296,2.8880551215,-1.6357465078\
C,0.2003525468,3.2120071528,-1.0729069365\C,-1.4307619656,1.1713036621,0.0196661096\
C,0.8784279284,1.8785415482,-0.7941986625\C,0.0938088997,0.9873346549,0.1797495184\
O,1.8943105009,1.5343110292,-1.3318205345\C,-1.707211341,-0.0165476655,-0.8655136037\
N,0.263072056, -0.4206224702,-0.1384701145\N,-0.7669589784,-0.8831379161,-0.8540530834\
C,-2.9952036185,-0.2953762279,-1.554229023\C,2.4388385003,-0.7018573827,0.8693819477\
C,1.3720686565,-1.2132823881,0.130767219\C,1.4128166649,-2.5386150265,-0.3063293461\
C,2.509069164,-3.32829272,-0.0071548324\C,3.5729125913,-2.8252775888,0.7301584857\
C,3.5255243522,-1.5087577524,1.164062534\H,-1.4734009836,3.3510921895,1.526604731\
H,-2.0006613324,4.5569355128,0.3208719763\H,-2.8719173499,2.5920949474,-0.7682455012\
H,0.78335513,3.8204135589,-1.7656944207\H,-1.9401647967,1.0073278989,0.9776573119\
H,0.4255530563,1.2186178987,1.1938998134\F,-3.0458591831,-1.5133555967,-2.0807167203\
F,-4.0261077942,-0.1730891228,-0.6959355171\F,-3.2294765699,0.5844029391,-2.5447691307\
H,2.4368023709,0.3274165283,1.2029460471\H,0.5845480971,-2.9366813801,-0.8759456084\
H,2.5277314218,-4.3544112907,-0.3565123959\H,4.4269396069,-3.450076557,0.9610606288\
H,4.3470221116,-1.0941759586,1.7369169739\\Version=ES64L-G16RevB.01\State=1-A\HF=-1174.0055261\ RMSD=6.740e-09\RMSF=2.885e-06\Dipole=-0.6892725,1.171306,1.8411607\
Quadrupole=-2.7097957,3.6174068,-0.9076112,-7.575362,-0.7869597,3.1712345\PG=C01
[X(C14H11F3N2O3)]\\@

TS-11'b
HF=-1173.8926305 a.u. (1, -278.2660 cm^{-1})
1\1\GINC-R09N40\FTS\RPBE1PBE\def2TZVP\C14H11F3N2O3\WURTHWE\28-Feb-2022
\0\\# pbe1pbe/def2tzvp opt=(ts,noeigentest,readfc) geom=check guess=read Pop=NBO Freq
emp=gd3bj scrf=(solvent=dichloromethane)\\Cycloaddition TS-11b-a\\0,1\
C,0.2812013955,0.7712324073,0.1053980945\C,0.3799197362,1.0582609557,1.4551736772\
C,1.2789621833,0.3442378315,2.2438545444\C,2.0862071026,-0.6451079199,1.6821976971\
C,1.9840543256, -0.9145145504,0.3292650854\C,1.0821445741, -0.212021926, -0.4614573606\
N,1.2929632702,0.6646911749,3.6071655196\C,-0.5936716554,-0.5137933747,4.7693611923\
C,0.0400664804,-0.941057257,5.9067314283\C,2.2022400557,-0.5849570745,5.3542656744\
N,2.079811528,0.0884435951,4.3583882079\C,-1.4356028089,0.674188339,4.828015243\
C,-1.5192434425,1.3029670001,6.2245978814\O,-1.5541134963,0.2956786186,7.1903700057\
C,-0.1915298055,-0.1548183072,7.1800928847\O,-0.3299083706,2.0377115592,6.4601246485\
C,0.5043961207,1.2044545082,7.2548737986\O,-2.0700102194,1.1275878744,3.896341592\
C,3.3974966636,-0.7596669483,6.227884746\F,4.2449910485,-1.6386082407,5.6922277203\
F,4.0667925462,0.3787718743,6.4358392152\F,3.021387285,-1.2237014199,7.4210213513\
H,1.517451959,1.2315418452,6.8591283661\H,0.5098563953,1.5592124656,8.2888652818\
H,-0.0262877493,-0.7662706667,8.0642977358\H,-2.3960591183,1.9458183768,6.3131744851\

H,0.346099931,-1.9742022447,6.0032730242\H,-0.655432459,-1.1306497864,3.8809859237\
H,-0.2404495456,1.813211687,1.9209696536\H,2.7924082284,-1.1872816742,2.3000965287\
H,2.6143328599,-1.6770586674,-0.1128595319\H,1.008355129,-0.428414069,-1.5204588592\
H,-0.4222803126,1.3204584308,-0.5087750718\\Version=ES64L-G16RevB.01\State=1-A\HF=-1173.8926305\RMSD=1.813e-09\RMSF=1.240e-06\Dipole=1.3434279,-1.3293536,-0.1931524\
Quadrupole=-11.4562372,-1.4697238,12.925961,0.8737573,-2.1712872,1.7001848\PG=C01 [X(C14H11F3N2O3)]
\\@

11'b-a

HF=-1174.0032162 a.u. (0)

1\1\GINC-R09N32\FOpt\RPBE1PBE\def2TZVP\C14H11F3N2O3\WURTHWE\28-Feb-2022\0\\# pbe1pbe/def2tzvp opt=readfc geom=check guess=read Pop=NBO Freq emp=gd3bj scrf=(solvent=dichloromethane)\\Product 11b-a\\0,1\
O,0.0333579719,2.878834184,0.242997286\C,-1.3375835301,2.5222158643,0.4302749184\
C,-1.8178110123,2.2040917002,-0.9805815621\O,-0.9802912033,3.0499393461,-1.7631538628\
C,0.2647554272,2.8960877082,-1.1375956016\C,-1.5939182028,0.7500363528,-1.4355008768\
C,0.825925868,1.5623070474,-1.6133281368\C,-0.1004883818,0.3559880547,-1.4462535065\
O,1.8863225951,1.4842357942,-2.1742309459\C,-2.1005567512,-0.2463972586,-0.4364663218\
N,0.0241643489,-0.3381407943,-0.1576736158\N,-1.1735514762,-0.7735357791,0.2655693772\
C,-3.5337758055,-0.5393590404,-0.1635519774\C,2.3038120543,-1.1114740472,-0.5015171494\
C,1.1692172651,-1.0075993579,0.296782186\C,1.1654239102,-1.5703931566,1.5736917505\
C,2.2870811802,-2.2332628874,2.0359132167\C,3.4263942275,-2.3372628687,1.2463787311\
C,3.4250595614,-1.7720207235,-0.0180430907\F,-4.1888316813,0.5703931349,0.234066068\
F,-4.1569232688,-0.9632306524,-1.2746697546\F,-3.7009984891,-1.4617729359,0.7762112836\
H,-1.4079016221,1.683630598,1.1228008447\H,-1.8763507713,3.3782145782,0.840508444\
H,-2.851984794,2.497067894,-1.1503171787\H,0.9326953943,3.7120911135,-1.4162065381\
H,-2.0342337193,0.63777786,-2.4259206946\H,0.1181905694,-0.3414419981,-2.2644697391\
H,2.3248518799,-0.6771436609,-1.4911445819\H,0.2804746558,-1.4855347349,2.1900304018\
H,2.2723335256,-2.6675393128,3.0289464893\H,4.30466157,-2.8519436488,1.6167311633\
H,4.3034127044,-1.845860373,-0.648792026\\Version=ES64L-G16RevB.01\State=1-A\HF=-1174.0032162\RMSD=2.631e-09\RMSF=2.625e-06\ Dipole=-0.4657534,0.2772102,-0.2568655\
Quadrupole=-2.5879746,-0.392283,2.9802576,-8.5297391,3.313754,0.5475758\PG=C01 [X(C14H11F3N2O3)]\\@

TS-11´b

HF=-1173.8975443 a.u. (1, -271.3116 cm^{-1})

1\1\GINC-R03N02\FTS\RPBE1PBE\def2TZVP\C14H11F3N2O3\WURTHWE\26-Feb-2022\0\\# pbe1pbe/def2tzvp Opt=(ts,noeigentest,readfc) geom=check guess=read Pop=NBO Freq emp=gd3bj scrf=(solvent=dichloromethane)\\Cycloaddition, TS-11´b\\0,1\ O,0.0248042801,-0.0527271074,-0.0780436975\C,0.0146100883,0.0051337312,1.340505369\
O,1.3494217812,0.035046243,1.740615538\C,1.9308253158,-0.9487245828,0.886943952\
C,1.3007569797,-0.5603933554,-0.4556268336\C,-0.6620539361,-1.2764498406,1.8276288716\
C,0.2271216896,-2.4433215083,1.8503904139\C,1.4837444478,-2.3099610584,1.3218851044\
O,-1.8491899789,-1.3064463175,2.0756506686\N,2.8105871059,-2.3466557166,3.2280742601\
N,1.8754531921,-2.3266585823,4.0389810262\C,0.6781433083,-2.283137397,4.1545625517\
C,-0.1800728027,-1.6954735568,5.2201035472\F,-1.3570723261,-2.3137480941,5.2769744929\
C,3.7813062959,-3.355257972,3.3342810138\C,4.866838346,-3.2685097849,2.4661772819\
C,5.8414900842,-4.2507106384,2.4855543872\C,5.7461416336,-5.3135429439,3.3746211761\
C,4.6664411797,-5.3909748007,4.2454661199\C,3.6801480115,-4.4201282434,4.2284149266\
F,0.3843872015,-1.7884855214,6.4260282365\F,-0.4105488807,-0.4043079977,4.9675563856\
H,1.1796440365,-1.404083557,-1.1362436795\H,1.8795312144,0.2300218583,-0.9398484406\
H,3.0115770964,-0.8353662891,0.9062090521\H,-0.5106482209,0.8970896162,1.683382924\
H,2.0423918012,-3.170342121,0.9738524981\ H,-0.2281691956,-3.4129552695,2.0060892693\
H,2.8382381192,-4.47949494,4.9080263561\H,4.5908770743,-6.2157445786,4.9443006228\

H,6.5139836067,-6.0775114806,3.3929509736\H,6.6840045956,-4.1824075253,1.8077340978\H,4.9351487961,-2.4284243181,1.7857067794\\Version=ES64L-G16RevB.01\State=1-A\HF=-1173.8975443\RMSD=3.318e-09\RMSF=1.204e-06\Dipole=3.0804598,-1.533678,-0.6341815\Quadrupole=-7.6278611,4.1989935,3.4288676,-1.3931157,-7.0537407,-1.8201026\PG=C01 [X(C14H11F3N2O3)]\\@

11´b

HF=-1174.0072863 a.u. (0)

1\1\GINC-R09N32\FOpt\RPBE1PBE\def2TZVP\C14H11F3N2O3\WURTHWE\08-Mar-202 2\0\\# pbe1pbe/def2tzvp Opt=readfc geom=check guess=read Pop=NBO Freq emp=gd3bj scrf=(solvent=dichloromethane)\\Product 11´b\\0,1\
C,0.9781675668,-0.4071235716,1.9554468472\C,0.9634857835,-0.115220261,3.4168948944\C,-0.250819355,0.278708366,4.2252413153\C,0.3735419659,0.3949491285,5.6381932417\N,1.7925817409,0.1472235983,5.3696390914\N,2.0418020843,-0.148249113,4.0966344594\C,-1.3931381713,-0.7218981782,4.0898547639\C,-1.6059614361,-1.6479366803,5.2785351766\O,-0.3902698393,-1.8706309795,5.9242682022\C,-0.180283257,-0.6423945431,6.6144244843\O,-2.4391344816,-0.9679186871,6.1896314607\C,-1.5794895769,-0.3119415302,7.1210387771\O,-2.0934755124,-0.7556463256,3.1144328278\C,2.8280264718,0.321371033,6.2790926502\H,-1.7876345595,0.7595730479,7.1331697663\H,-1.7513666455,-0.7282383131,8.1148317691\H,0.5292930027,-0.8266112179,7.418617949\H,-2.0589850983,-2.591327276,4.9720557554\H,0.2357060192,1.3998664377,6.0447729946\H,-0.6393395194,1.2347708685,3.8661891892\C,4.1264126591,-0.0820413906,5.9570008133\C,5.1486517192,0.0953038808,6.8706357683\C,4.9062518848,0.6666211927,8.1143678616\C,3.6180459491,1.0672652273,8.4295229259\C,2.5805736839,0.9019010775,7.5239306899\H,4.3190496173,-0.5287613197,4.9912951806\H,6.1506305135,-0.2228464834,6.6061452587\H,5.7122937447,0.7976089004,8.8257303293\H,3.4079658506,1.5209209849,9.3911898372\H,1.5884603175,1.2404574,7.7928278607\F,0.4282612228,-1.6011223096,1.6739578899\F,0.2722281132,0.5101106012,1.2775627396\F,2.213940422,-0.4179953574,1.4608249402\\Version=ES64L-G16RevB.01\State=1-A\HF=-1174.0072863\RMSD=3.595e-09\RMSF=2.923e-06\Dipole=0.2408174,1.1209976,2.6640801\Quadrupole=-3.2840916,2.9598339,0.3242577,-3.2399788,-4.4638116,2.6213419\PG=C01 [X(C14H11F3N2O3)]\\@

TS-11´b-a

HF=-1173.8924592 a.u. (1, -301.2455 cm^{-1})

1\1\GINC-R08N47\FTS\RPBE1PBE\def2TZVP\C14H11F3N2O3\WURTHWE\28-Feb-2022 \0\\# pbe1pbe/def2tzvp opt=(ts,noeigentest,readfc) geom=check guess=read Pop=NBO Freq emp=gd3bj scrf=(solvent=dichloromethane)\\Cycloaddition TS-11´b-a\\0,1\
C,0.0420592228,0.307944171,0.1592549729\C,-0.1960276523,0.0612317364,1.509809009\C,0.7876464131,-0.5381254286,2.2971902171\C,1.9988708911,-0.8876980596,1.7258799814\C,2.2360649789,-0.6523479465,0.3775027862\C,1.2535438353,-0.05428969,-0.4018973134\N,-1.4600361258,0.4177313458,1.9947480587\C,-3.5739204081,-0.6333734087,1.2326520093\C,-4.2204338176,-0.5882276572,2.4397565984\C,-2.5936816091,-0.1885246656,3.9299666542\N,-1.7307963752,0.2145914368,3.1876677356\C,-4.7126920561,-1.856322028,3.0135037541\C,-4.2833783256,-3.0981975449,2.2242256199\O,-4.3877317525,-2.8422261879,0.8580592619\C,-3.2581864464,-1.9779309726,0.6411203733\C,-2.1915271183,-2.7776788125,1.3900540141\O,-2.9123464238,-3.3294272288,2.4872911835\O,-5.4190923346,-1.9349616399,3.9918691094\C,-2.9467184091,0.2240537145,5.3177322783\F,-1.961261999,0.9010955801,5.9131116193\F,-4.0304993162,1.0039018835,5.2991868428\F,-3.2191573569,-0.8340948501,6.0780098914\H,-4.8863796864,-3.9647970482,2.4985072743\H,-1.8167026323,-3.5828040043,0.7530079032\H,-3.073966867,-1.9191822111,-0.4289190674\H,-1.357569545,-2.1872453512,1.7605909876\H,-3.4944562989,0.2432447718,0.6083337354\H,-4.7112511659,0.319644258,2.7757708872\H,0.6020826514,-0.7182736037,3.3496894452\H,2.7641458366,-1.3484520374,2.3394003539\H,3.1856485606,-0.9305570069,-0.0633629849\H,1.4341397553,0.1345954019,-1.4534313474\

H,-0.7338314476,0.775187817,-0.435369159\\Version=ES64L-G16RevB.01\State=1-A\ HF=-1173.8924592\RMSD=6.322e-09\RMSF=4.245e-06\Dipole=2.1394664,0.5936142,-2.2239096\ Quadrupole=-0.1997606,-2.6062214,2.8059821,-5.8482067,1.8395127,-0.3857228\PG= C01 [X(C14H11F3N2O3)]\\@

11´b-a

HF=-1174.0082145 a.u. (0)

1\1\GINC-R09N17\FOpt\RPBE1PBE\def2TZVP\C14H11F3N2O3\WURTHWE\28-Feb-202
2\0\\# pbe1pbe/def2tzvp opt=readfc geom=check guess=read Pop=NBO Freq
emp=gd3bj scrf=(solvent=dichloromethane)\\Product 11´b-a\\0,1\
C,0.0434962098,0.0443875654,-0.0243985608\C,0.0006908429,0.0666932982,1.49627405\
O,1.3203027323,0.0917306401,1.964651752\C,0.4365272744,2.185610923,2.214309274\
C,1.6924681673,1.4517380571,1.7706123427\C,2.0558288876,1.6355196229,0.2877363075\
C,0.9743653621,1.0688147732,-0.6555348684\O,-0.5951941393,-0.7293558167,-0.6853945782\
H,-0.5255484737,-0.8039542806,1.8891389253\H,0.4529040764,2.3694640796,3.2900654187\
H,2.5481167858,1.6647489133,2.4080338343\H,0.2640594394,3.1250820146,1.6891220563\
H,3.0173942489,1.1568992558,0.1035785519\H,1.4132289547,0.5850421335,-1.5344046251\
N,2.0844631101,3.0428430078,-0.1023797026\C,2.9829743732,3.9983260046,0.3604282732\
N,0.9950694282,3.3958049175,-0.7894829694\C,0.3142326193,2.3572735103,-1.0876881495\
C,-0.9334658668,2.4637820122,-1.8951165869\O,-0.6104531155,1.2615302579,1.9124638347\
F,-1.1777047009,3.7122377586,-2.2817146381\F,-0.8576408311,1.6995805061,-2.9966658253\
F,-2.0046762688,2.0365347378,-1.2067759197\C,2.7484375381,5.3579963186,0.1463459191\
C,3.6616183853,6.2920386901,0.6015795653\C,4.812706648,5.899509354,1.2731804024\
C,5.0419272108,4.5483625486,1.4811305525\C,4.1395159073,3.5978645168,1.0299960479\
H,1.8532182205,5.6679939863,-0.3747047073\H,3.4662934926,7.3443511055,0.4296501156\
H,5.5208457576,6.6379657737,1.6282628178\H,5.9358709551,4.2200936101,1.9985525224\
H,4.3504806426,2.5486167418,1.1919390997\\Version=ES64L-G16RevB.01\State=1-A\
HF=-1174.0082145\RMSD=4.230e-09\RMSF=3.904e-06\Dipole=2.2412855,0.496704,1.3559735\
Quadrupole=-0.3155234,-0.3329872,0.6485106,-4.0579485,-0.9499066,-0.2212226\PG=C01
[X(C14H11F3N2O3)]\\@

Section 6: *References*

[1] CrysAlisPRO software system, Oxford Diffraction/Agilent Technologies UK Ltd, Yarnton, England, 2015.

[2] Dolomanov, O. V.; Bourhis, L. J.; Gildea, R. J.; Howard, J. A. K.; Puschmann, H. J. *OLEX2*: a complete structure solution, refinement and analysis program. *Appl. Cryst.* **2009**, *42*, 39–341. DOI: org/10.1107/S0021889808042726.

[3] Sheldrick, G. M. *SHELXT* - Integrated space-group and crystal-structure determination *Acta Cryst.* **2015**, *A71*, 3–8. DOI: org/10.1107/S2053273314026370.

[4] Sheldrick, G. M. A short history of *SHELX*. *Acta Cryst.* **2008**, *A64*, 112–122. DOI: org/10.1107/S0108767307043930.

[5] Gaussian 16, Revision B.01, Frisch, M. J.; Trucks, G. W.; Schlegel, H. B.; Scuseria, G. E.; Robb, M. A.; Cheeseman, R.; Scalmani, G.; Barone, V.; Petersson, G. A.; Nakatsuji, H.; Li, X.; Caricato, M.; Marenich, A. V.; Bloino, J.; Janesko, B.G.; Gomperts, R.; Mennucci, B.; Hratchian, H. P.; Ortiz, J. V.; Izmaylov, A. F.; Sonnenberg, J. L.; Williams-Young, D.; Ding, F.; Lipparini, F.; Egidi, F.; Goings, J.; Peng, B.; Petrone, A.; Henderson, T.; Ranasinghe, D.; Zakrzewski, V. G.; Gao, J.; Rega, N.; Zheng, G.; Liang, W.; Hada, M.; Ehara, M.; Toyota, K.; Fukuda, R.; Hasegawa, J.; Ishida, M.; Nakajima, T.; Honda, Y.; Kitao, O.; Nakai, H.; Vreven, T.; Throssell, V; Montgomery, Jr., J. A.; Peralta, J. E.; Ogliaro, F.; Bearpark, M. J.; Heyd, J. J.; Brothers, E. N.; Kudin, K. N.; Staroverov, V. N.; Keith, T. A.; Kobayashi, R.; Normand, J.; Raghavachari, K.; Rendell, A. P.; Burant, J. C.; Iyengar, S. S.; Tomasi, J.; Cossi, V; Millam, J. M.; Klene, M.; Adamo, C.; Cammi, R.; Ochterski, J. W.; Martin, R. L.; Morokuma, K.; Farkas, O.; Foresman, J. B.; Fox, D. J. Gaussian, Inc., Wallingford CT, 2016.

[6] Becke, A. D. Density-functional thermochemistry. III. The role of exact exchange. *J. Chem. Phys.* **1993**, *98*, 5648–5652. DOI: 10.1063/1.464913.

[7] Lee, C.; Yang, W.; Parr, R. G. Development of the Colle-Salvetti correlation-energy formula into a functional of the electron density. *Phys. Rev. B* **1988**, *37*, 785–789. DOI: 10.1103/physrevb.37.785.

[8] Grimme, S.; Ehrlich, S.; Goerigk, L. Effect of the damping function in dispersion corrected density functional theory. *J. Comput. Chem.* **2011**, *32*, 1456–1465. DOI: org/10.1002/jcc.21759.

[9] Grimme, S.; Hansen, A.; Brandenburg, J. G.; Bannwarth C. Effect of the damping function in dispersion corrected density functional theory. *Chem. Rev*. **2016**, *116*, 5105–5154. DOI: org/10.1021/acs.chemrev.5b00533.

[10] Perdew, J. P.; Burke, K.; Ernzerhof, M. Generalized Gradient Approximation Made Simple. *Phys. Rev. Lett.* **1996**, *77*, 3865–3868. DOI: org/10.1103/PhysRevLett.77.3865.

[11] Perdew, J. P.; Burke, K.; Ernzerhof, M. Generalized Gradient Approximation Made Simple [Phys. Rev. Lett. 77, 3865 (1996)]. *Phys. Rev. Lett.* **1997**, *78*, 1396. DOI: org/10.1103/PhysRevLett.78.1396.

[12] Adamo, C.; Barone, V. Toward reliable density functional methods without adjustable parameters: The PBE0 model. *J. Chem. Phys.* **1999**, *110*, 6158–6169. DOI: org/10.1063/1.478522.

[13] Ernzerhof, M.; Scuseria, G. E. Assessment of the Perdew–Burke–Ernzerhof exchange-correlation functional. *J. Chem. Phys.* **1999**, *110*, 5029–5036. DOI: org/10.1063/1.478401.

[14] Weigend, R.; Ahlrichs, R. Balanced basis sets of split valence, triple zeta valence and quadruple zeta valence quality for H to Rn: Design and assessment of accuracy. *Phys. Chem. Chem. Phys.* **2005**, *7*, 3297–3305. DOI: 10.1039/B508541A.

[15] Tomasi, J.; Mennucci, B.; Cammi, R. Quantum Mechanical Continuum Solvation Models. *Chem. Rev.* **2005**, *105*, 2999–3093. DOI: org/10.1021/cr9904009.

Section 7: *List of captions for schemes and figures of the main manuscript*

Scheme 1. Generation of fluorinated nitrile imines **1** and two literature examples of their (3+2)-cycloadditions with chalcone **2** and thiochalcone **4**, leading to pyrazoline **3** and 1,3,4-thiadiazoline derivative **5**, respectively [7,14].

Figure 1. Levoglucosenone (**6**), its stereochemical structure, and selected products of cycloaddition reactions with a *C*(Ph),*N*(Ph) nitrile imine (**9**) and tropothione lead to polycylic, isomeric pyrazoles **7**/**7′** [30] and polycylic thiophene derivative **8** [39], respectively. In structures **7**/**7′**, the skeleton of *C*(Ph),*N*(PH) nitrile imine **9** is presented in red.

Scheme 2. Selectivity was observed in the (3+2)-cycloaddition of *C*(Ph),*N*(Ph) nitrile imine (9) [7,30] with levoglucosenone (6) in THF solution at room temperature, providing pyrazolines *exo*-10 and *exo*-10′ as primary products, followed by oxidation to isolated pyrazole derivatives 7 and 7′.

Figure 2. The molecular structure of polycylic pyrazole **7** was estimated by means of a single crystal X-ray experiment. Atoms are represented by thermal elipsoids (50%) for clarity.

Figure 3. Molecular structure of polycylic pyrazoline *exo*-**11a**, estimated by means of a single crystal X-ray experiment. Atoms are represented by thermal elipsoids (50%) for clarity.

Scheme 3. Highly selective (3+2)-cycloadditions of fluorinated nitrile imines **1a**–**1i**, derived from trifluoroacetonitrile, with levoglucosenone (**6**) leading to trifluoromethyl substituted, fused pyrazolines **11a**–**11i**.

Scheme 4. Equation above: spontaneous oxidation of pyrazoline *exo*-**11i** with air oxygen during chromatographic purification, leading to the fused pyrazole **12a**. Equation below: dehydrogenation of pyrazoline *exo*-**11g** using MnO_2 as an oxidizing reagent, leading to the fused pyrazole **12b**.

Scheme 5. Attempted oxidation of pyrazoline *exo*-**11g** with TCCA, leading to a mixture of fused pyrazole **12b** and its chlorinated derivative **12c**.

Figure 4. Competitive *exo*- and *endo*-approaches of nitrile imines **1** onto the enone fragment of levoglucosenone **6** in the transition state of the (3+2)-cycloaddition reaction.

Figure 5. DFT calculations of the (3+2)-cycloadditions of trifluoromethyl-substituted nitrile imine **1b** with levoglucosenone **6** (PBE1PBE/def2tzvp + GD3BJ +PCM-dichloromethane) [kcal/mol] led to the regioisomeric pyrazolines *exo*-**11b**/*endo*-**11b** and *exo*-**11′b**/*endo*-**11′b**. Symbol‡: transition state.

Figure 6. Calculated atomic distances of the forming bonds of the transition states **TS**-*exo*-**11b** and **TS**-*exo*-**11′b** (PBE1PBE/def2tzvp + GD3BJ +PCM-dichloromethane). (**a**): **TS**-*exo*-**11b** atomic distances C–N 2.569 Å, C–C 2.253 Å; (**b**): **TS**-*exo*-**11′b** atomic distances C–N 2.323 Å C–C 2.353 Å.

Figure 7. DFT calculations of the cycloadditions of diphenyl-substituted nitrile imine **9** with levoglucosenone **6** (PBE1PBE/def2tzvp + GD3BJ +PCM-dichloromethane, room temperature) [kcal/mol] led to diastereomeric and regioisomeric pyrazolines *exo*/*endo*-**10** and *exo*/*endo*-**10′**. Symbol‡: transition state.
